# Interface Evolution and Long-Term Performance of Negative Carbon Fiber Structural Electrodes

**DOI:** 10.1021/acsomega.5c01630

**Published:** 2025-07-01

**Authors:** Lynn Maria Schneider, Benedikt Sochor, Marcus Johansen, Fang Liu, Göran Lindbergh, Dan Zenkert, Stephan Roth, Sarathlal Koyiloth Vayalil, Louise Lebret

**Affiliations:** † Department of Engineering Mechanics, 7655 KTH Royal Institute of Technology, Stockholm SE-100 44 , Sweden; ‡ 28332 Deutsches Elektronen-Synchrotron DESY, Notkestr. 85, Hamburg 22607, Germany; § Advanced Light Source, Lawrence Berkeley National Laboratory, Berkeley, California 94720 United States; ∥ Department of Industrial and Materials Science, 11248 Chalmers University of Technology, Gothenburg SE-412 96, Sweden; ⊥ Department of Chemical Engineering, KTH Royal Institute of Technology, Stockholm SE-100 44, Sweden; # Department of Fibre and Polymer Technology, KTH Royal Institute of Technology, Stockholm SE-100 44, Sweden; ∇ Applied Science Cluster, UPES, Dehradun, Uttarakhand 248007, India

## Abstract

Laminated structural batteries present a transformative solution to reducing weight constraints in electric vehicles. These structural batteries are based on a multifunctional material that incorporates an energy storage function within a carbon fiber-reinforced polymer. Despite the potential of this technology, the intricate morphology of fiber–matrix or electrode–electrolyte interfaces and the impact of long-term cycling at low current rates (C-rates) on these interfaces remain insufficiently understood. This study addresses these critical knowledge gaps by examining the influence of matrix composition on the long-term electrochemical performance of structural battery electrodes and exploring advanced techniques to investigate carbon fiber–matrix interfaces. Localized imaging and X-ray scattering techniques were used to characterize morphological changes at the electrode–electrolyte interfaces by analyzing negative structural electrodes. The findings revealed that the matrix composition influences long-term electrochemical behavior and fiber–matrix interface formation. While the intrinsic properties of carbon fibers largely remain unaffected by long-term cycling, cycling promotes debonding at fiber–matrix interfaces. Nonetheless, residual regions of adhesion persist, underscoring the potential for preserving multifunctionality even under prolonged cycling conditions. These insights advance the understanding of interface dynamics, which is critical for optimizing structural battery technologies.

## Graphical abstract

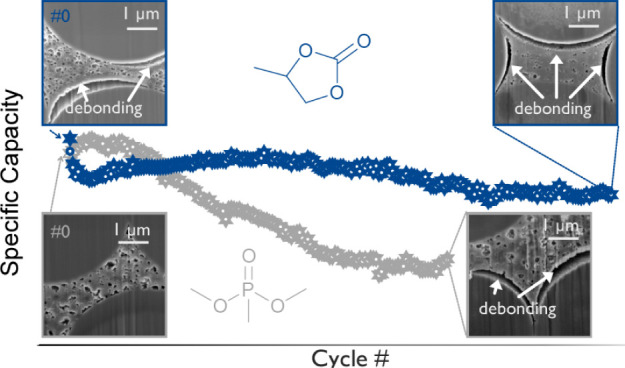

## Introduction

Laminated structural batteries can be an effective way to reduce the weight of electric vehicles and therefore increase their range.^1,2^ Structural batteries consist of a lightweight carbon fiber (CF)-reinforced polymer, which provides load-bearing properties at low mass.^3^ Simultaneously, the specific design of this composite material enables an energy storage function, making this structural battery a multifunctional material. In the laminated structural battery design, CFs act as electrodes and reinforce the material. The negative electrode uses commercially available CFs that function as intercalation electrodes for lithium ions (Li ions), similar to graphite, and they conduct electrons.^4^ The positive electrode uses CFs coated with electrochemically active material, such as LiFePO_4_, while CFs also act as current collectors.^5,6^ The electrodes are separated by a glass fiber separator, and the individual constituents are impregnated with a multifunctional matrix. As a matrix material, a bicontinuous electrolyte with a solid, porous polymer backbone and a percolating liquid electrolyte phase is often used.^7–9^ This bicontinuous electrolyte can be made by using a process called polymerization-induced phase separation. In this process, a mixture of monomer and liquid electrolyte is initially miscible. The polymerization of the monomer then leads to a phase- separation of polymer from the liquid electrolyte due to differences in solubilities.^7,8^ The liquid phase then enables ion transport, and the solid polymer phase transfers mechanical loads between the fibers, making it a multifunctional matrix.

The interface between the CFs and the multifunctional matrix (i.e., structural electrolyte) is a key junction for achieving and optimizing the multifunctional performance of structural battery laminates.^10,11^ This interface must transfer mechanical loads through the solid polymer’s structural network while enabling Li-ion transport via the percolating liquid electrolyte. In a structural battery, both aspects must be met to ensure balanced performance in both functions. From a composite material perspective, fiber–matrix adhesion is particularly important for achieving a functional composite structure. From a battery perspective, the accessibility of the fibers and efficient ion transport across the interface are of utmost importance, as limitations in these areas can reduce rate performance or even diminish charging capacities. Therefore, both the solid and liquid components of the bicontinuous electrolyte must be in contact with the fiber surface.^10^

Previous studies have shown that CFs in the negative electrode expand upon lithiation (i.e., Li-ion intercalation), amounting to 8–13% radial and 1% longitudinal expansions.^12^ This expansion is proportional to the number of Li ions intercalated (i.e., specific capacity), which is typically higher for CFs at low current densities. The expansion leads to two challenges in battery design. On one hand, the polymer part of the multifunctional interface must withstand these periodic strains upon cycling. On the other hand, the effect of these expansions on the electrochemical long-term properties remains unknown. Repeated expansions and contractions can lead to degradation of the liquid electrolyte, as active Li ions react with freshly exposed electrode surfaces during cycling. Numerical studies on a single-fiber microbattery have shown that these expansions initiate and grow microcracks from the fiber–matrix interface.^13,14^ The adhesion between the CFs and the matrix is directly affecting the transverse mechanical properties of a unidirectional (UD) CF lamina.^15^ In our most recent work,^16^ we found that the transverse stiffness of the UD all-fiber structural battery decreases after electrochemical cycling. Xu et al.^10^ showed that different structural electrolyte (SE) formulations lead to changes in adhesive properties between fibers and the matrix. Our previous work^9^ also demonstrated that the SE formulation influences the electrochemical cycling performance independently of its bulk properties, potentially due to variations in CF–matrix interface formation. The addition of thiols can increase the adhesive properties between CFs and the matrix, a SE composition introduced in our previous work.^9^ However, previous work^9^ lacks long-term cycling, interfacial morphology evaluation, and mechanical property assessments of different SE compositions.

To summarize, there is a lack of nondestructive experimental techniques to study and understand multifunctional interfaces in structural batteries. Furthermore, current long-term performance studies report data for 1000 cycles obtained at high current densities (∼136 mA g^–1^), which limit dimensional CF expansion and are often conducted in only liquid electrolytes.^17^

The present study focuses on investigating the long-term properties of structural electrodes and their impact on the CF–matrix interfaces. The study investigates how different SE formulations influence the CF–matrix interface morphology and how these SE formulations accommodate the cyclic strains caused by long-term charging and discharging by using low C-rates. Advanced experimental techniques were used to reveal the morphological changes at the interfaces. Negative structural electrodes with different SE formulations were electrochemically and mechanically evaluated before and after long-term cycling (100–200 cycles at 18.6 mA g^–1^). Structural electrodes with selected SE formulations from previous work^8,9^ were evaluated for differences in long-term stability, elastic modulus, capacity fade, and electrode–electrolyte interface morphology. Morphology was investigated using a combination of synchrotron small-angle X-ray scattering (SAXS) as a nondestructive technique and focused ion-beam scanning electron microscopy (FIB-SEM), as well as cryogenic scanning electron microscopy (cryo-SEM). Furthermore, the impact of drying (prior to SEM analysis) on the porous SE morphology was revealed. The findings demonstrate that modifications to the SE formulation can alter the interfacial properties of the structural electrodes. Furthermore, it is observed that even after prolonged electrochemical cycling, segments of the SE polymer remain in contact with the CFs, a critical factor for maintaining the high-performance characteristics of the composite material.

## Methodology

### Materials

The monomer used in this study was bisphenol A ethoxylate dimethacrylate (BPAEDM) (*M*_n_ = 540 g/mol) and was supplied by Sartomer Company, Europe. Dimethyl methylphosphonate (DMMP, 97%), propylene carbonate (PC, 99% anhydrous), ethylene carbonate (EC, 99% anhydrous), lithium trifluoromethanesulfonate (LiTFS) (96%), lithium bis­(trifluoromethanesulfonyl)­imide (LiTFSI, 99.95%), and 2,2′-azobis­(2-methylpropionitrile) (AIBN) were obtained from Merck. T800S CFs (12k) from Toray were provided as 17-mm spread tows from Oxeon AB. For current collection, copper (17 μm, 99.95% purity) and nickel foil (15 μm, 99.95%) were obtained from Advent Research Material Ltd. The pouch material PET/Al/PE from Skultuna Flexible was used. For half cells, lithium metal (380 μm, 99.9% purity) and 260-μm-thick Whatman GF/A glass fiber paper were obtained from Sigma-Aldrich and used as received. For mechanical testing, DeltaPrepreg (W105P-DT806W, Toray) was used as tabbing material, and a two-component epoxy (EA9466, Loctite) was used as adhesive.

### Structural Electrode Manufacturing

#### Structural Electrode and Half-Cell Preparation

The UD structural electrodes and negative half-cells were manufactured using a process similar to that described by Schneider et al.^8^ The spread T800S CF tow was taped onto a glass plate (17 × 140 mm). A copper foil current collector (5 × 70 mm) was placed across the width of the CF tow and attached using Electrolube silver conductive paint. The CF tow and glass plate were then enclosed in a vacuum infusion bag, which was sealed with an additional outer bag to enhance vacuum integrity and prevent leaks. The entire assembly was dried in a vacuum oven at 60 °C for 24 h. It should be noted that the vacuum bags were cut open and resealed for the drying process. Then, the resin mix was prepared inside a glovebox (<4 ppm of H_2_O, <4 ppm of O_2_). Two stock solutions of the liquid electrolyte were made for DMMP and PC-based samples, respectively. The DMMP samples contained 39% of DMMP/EC (50:50 wt) with 1 M LiTFSI. The PC samples contained 39% of PC/EC (50:50 wt) with 1 M LiTFSI. For the samples containing vinylene carbonate (VC), an amount of 1 wt % based on the total amount of liquid electrolyte was incorporated into the liquid electrolyte prior to curing. The respective SE formulations were prepared in a glass vial where the monomer was mixed with the respective liquid electrolyte (39 wt %) and thermal initiator AIBN (1 wt % of monomer weight) until a homogeneous solution was obtained. The glass vial was then sealed with a septum, and the glass plate with CFs was then vacuum-infused with the selected SE formulation outside the glovebox. All samples were cured at 90 °C for 45 min.

After curing, the structural electrode was moved to the glovebox, and the half-cells were then assembled in a dry argon atmosphere (<4 ppm of H_2_O, < 4 ppm of O_2_). The structural electrodes were cut into 40-mm-long stripes and layered with a Whatman glass–microfiber filter separator and polished lithium foil as a counter electrode combined with a nickel current collector.

A small volume of liquid electrolyte (200 μL), with the compositions listed in Table 1, was added to the separator of each respective sample to ensure complete wetting. Next, the pouch cells were sealed and electrochemically tested. For parts of this study, samples manufactured in a previous study^9^ were reused for continued cycling. These samples were used to evaluate the long-term stability during continued cycling (100 cycles in total). The manufacturing of these samples can be found elsewhere.^9^ The nomenclature of the samples with different SE formulations can be found in Table 1. In general, the first part of the sample name indicates a variation in either porogen structure, monomer composition, or electrolyte composition. The first number typically describes the amount of liquid electrolyte (wt %) applied in the matrix in comparison to the solid counterpart. Most of the studied formulations are based on a PC/EC 1 M LiTFSI liquid electrolyte.

**Table 1:** Nomenclature for Samples, Defining Electrolyte Composition and Content, and Monomer Composition

Name	Liquid Electrolyte Composition	Liquid Electrolyte Content	Monomer Composition
DMMP39	DMMP/EC 1 M LiTFS	39%	BPAEDM
PC39	PC/EC 1 M LiTFSI	39%	BPAEDM
PCVC39	PC/EC 1 M LiTFSI, 1 wt % of VC	39%	BPAEDM
PC50	PC/EC 1 M LiTFSI	50%	BPAEDM
Thiol50	PC/EC 1 M LiTFSI	50%	BPAEDM and thiol^a^
PC503M	PC/EC 3 M LiTFSI	50%	BPAEDM
PC10	PC/EC 1 M LiTFSI	10%	BPAEDM

a The thiol used was dipentaerythritol hexakis­(3-mercaptopropionate) with 2.36% of total monomer weight.

### Electrochemical Analysis

All samples were galvanostatically cycled using a Neware battery cycler (CT-4008-5V10mA-164) or a Biologic potentiostat in a room conditioned to 23 °C and 50% relative humidity. The half-cells, using lithium as a counter electrode, were galvanostatically cycled for a total of 100–200 cycles, applying a current rate of 18.6 mA g^–1^. The cells were cycled between 0.002 and 1.5 V vs Li^+^ at ambient temperature. For the samples cycled for 100 cycles (i.e., PC39, PC50, Thiol50, PC503M), the cells had current collectors attached to both sides of the electrode. These samples were precycled in a previous study^9^ at different current densities with 18.6 mA g^–1^ for 5 cycles, 37.2 mA g^–1^ for 5 cycles, 74.4 mA g^–1^ for 20 cycles, and 18.6 mA g^–1^ for 10 cycles. In this study, these samples were exposed to continued cycling for 60 cycles at 18.6 mA g^–1^. The capacities were estimated based on the linear densities of the T800S CF (0.52 g m^–1^). For the first cycle losses, the first lithiation was related to the first delithiation. For the capacity retention calculations, the fifth cycle was taken as the reference capacity to ensure a few formation cycles. For the half-cells with 140 cycles or more, at least three cells were manufactured and tested.

### Focused-Ion Beam Scanning Electron Microscopy

For FIB-SEM, the samples were first soaked in deionized water for 24 h to extract the liquid electrolyte and subsequently dried for 12 h in a vacuum oven at 60 °C. The degree of drying was gravimetrically evaluated. A Tescan Gaia 3 FIB-SEM instrument was used. It is equipped with a gas injection system that allows in situ deposition of a Pt layer (2 μm thick) to cover and protect the region of interest and mitigate the curtaining artifact. The ion column was operated at an accelerating voltage of 30 kV. For the coarse milling stage, a beam current of 42 nA was applied with a milling time of around 20 min. A staircase milling pattern and a trench size of 30 × 30 μm were used. For the fine milling, a beam current of 2.6 nA was used with a rectangular milling pattern directed from the trench toward the region of interest in order to mitigate redeposition. The milling area size was 30 μm × 0.5 μm with an approximate milling time of around 5 min.

### Cryogenic Scanning Electron Microscopy

Cryo-SEM was applied to investigate the samples in their wet state without drying and extracting a liquid electrolyte. For the cryoanalysis, a JSM-IT800 instrument with a Leica cryo-stage was used. The samples were immersed in a nitrogen slush to ensure fast freezing rates. The frozen samples were freeze-cut or freeze-fractured and transferred to the cryo-stage in SEM using a Leica EM VCT500. Secondary electron images were recorded under cryo-condition with the sample stage at −100 °C and an accelerating voltage of 5 kV.

### Synchrotron Small-Angle X-ray Scattering

For SAXS experiments, the samples were first dried in the same way as described in the previous section for SEM preparation. Next, cross-sectional electrode samples (∼0.08 mm wide, 8 mm long, and 3 mm thick) were cut and mounted onto a 2-mm wide Kapton tape (50 μm thick). The cross-sectional samples were then fixed on a frame with Kapton tape. The frame was then placed in the synchrotron beam and measured in the fiber direction with the cross-section facing the beam with an exposure time of 1 s. SAXS experiments were conducted at beamline P03 (PETRA III, DESY, Hamburg, Germany).^18^ The beam size, wavelength, and sample-to-detector distance were fixed at (*H* × *V*) 30 × 30 μm^2^, 1.023 Å (*E* = 12.12 keV), and 9330 mm for the microfocused X-ray beam. The scattered photons were collected using a PILATUS 2 M detector (pixel size = 172 μm, Dectris, Baden, Switzerland) for the microfocus experiments. The collected 2D scattering patterns were azimuthally integrated and scaled to absolute intensities using the standard procedures in the literature.^19–21^ The data were presented as normalized with thickness (i.e., 3-mm fiber length). Four line scans were performed across the specimen width with a 100 μm spacing between each line scan. The highest-intensity images from each line scan were summed and are shown for both uncycled and cycled dried structural electrodes.

### Mechanical Testing

Tensile tests were conducted on virgin CF, cycled, and uncycled structural electrodes. Before testing, the samples were cut into 22 × 3 mm sections and tabbed using glass fiber tabs (10 mm × 12 mm) with two-component Loctite adhesive. Five mm of each sample side was adhered to the tabs and cured overnight (∼12 h) with an additional postcuring step of 4 h at 50 °C. The final testing specimen thus had a span of 12 mm and a width of 3 mm and was tested in the fiber direction (longitudinal). It should be noted that the specimen size deviates from the standard (D5083 ASTM) recommended ratio of 10:1, while the used specimen size results in a ratio of 4:1, but the limitations were due to the available specimen material and manufacturing capabilities. However, apart from the sample size, all of the general guidelines of the standard were respected. An ElectroForce DMA3200 from TA Instruments with a 500 N load cell in ramp mode and a test speed of 0.001 mm s^–1^ was used for the tensile test. At least five specimens were tested for each sample type. The expected modulus was calculated based on the individual thicknesses and fiber volume fractions.

## Results and Discussion

This study explores the electrochemical and mechanical longevity of structural electrodes with selected SE compositions (Table 1). The primary focus of this study is the influence of porogen structure on the long-term performance of structural electrodes with PC39 and DMMP39 samples evaluated over 200 cycles using FIB-, cryo-SEM and SAXS analyses. Notably, previous findings^9^ indicated conflicting behavior between bulk SE properties and structural electrode performance when considering a change in porogen structure. A structural electrode with an SE composition using a well-known electrolyte additive, PCVC39, was assessed for its potential to reduce capacity fade. Additionally, the study electrochemically evaluated various SE compositions over 100 cycles to assess: (i) whether increased salt concentration can increase capacity retention (PC503M), (ii) whether higher porogen content mitigates capacity fade through excess liquid electrolyte (PC50) and (iii) whether incorporation of thiol enhances the fiber-matrix adhesion in structural electrodes (Thiol50).

### Long-Term Cycling of Structural Electrodes

Figure 1 shows the specific capacities for structural electrodes with SE formulations PC39, DMMP39, and PCVC39 with lithium foil as the counter electrode charged and discharged over 200 cycles at 18.6 mA g^–1^. It is noteworthy that replicate samples can differ in terms of specific capacities and capacity retention due to lab-manufactured cells (see Table S1 and Figures S2–S4). However, general overall trends can still be deduced.

**Figure 1:**
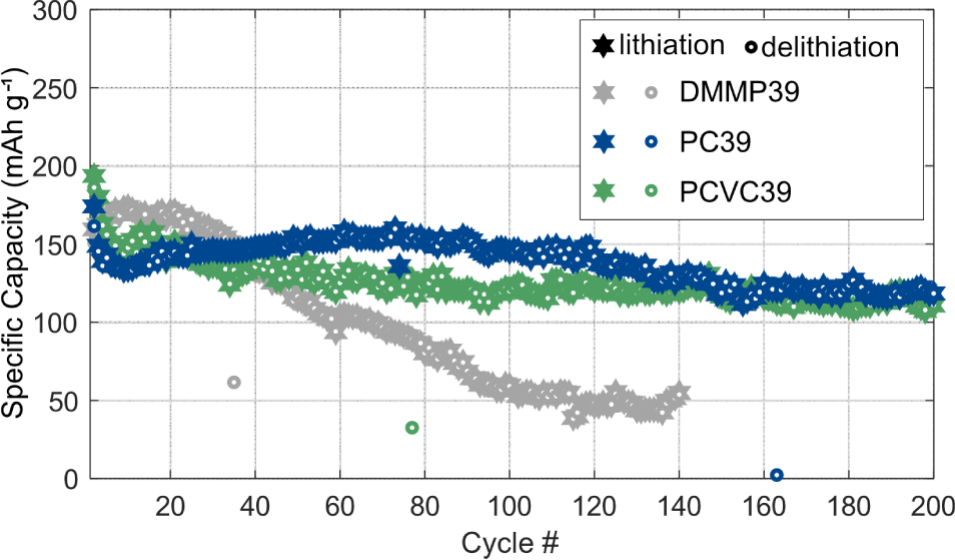
Capacity vs cycle number with a current density of 18.6 mAh g^–1^ for 200 cycles of structural electrodes with different SE formulations (DMMP39, PC39, PCVC39). The calculated capacity retentions and first cycle losses for the different samples can be found in Table S1. The shown data refer to different samples in Table S1: DMMP39 shows sample S2; PC39 shows sample S1; and PCVC39 shows sample S1.

The results show that the PC39-based formulation without additives outperforms all samples, with an average capacity retention of 79% after 200 cycles (Table S1). The corresponding Coulombic efficiency data (Figure 2d) is stable at around 100% after the first few cycles, which is in accordance with previous work.^22^ These data confirm the reversible intercalation of Li ions and indicate the absence of major side reactions for the PC39 samples. The fluctuations in Coulombic efficiency around 100% can be related to Li-ion accessibility slightly fluctuating between cycles. The image of the PC39 structural electrode shows a yellow discoloration of the separator, where the current collector was adhered to the structural electrode (Figure S5d). This could indicate that the adhesive in the current collector may have caused an unwanted side reaction, leading to capacity fade or corrosion of the current collector, which increases the internal resistances.^23^ The first cycle losses of the PC39 samples average around 30% (Table S1), which is observed in CF literature and associated with trapped lithium in the amorphous region of the fibers and solid electrolyte interface (SEI) layer formation.^24^

**Figure 2:**
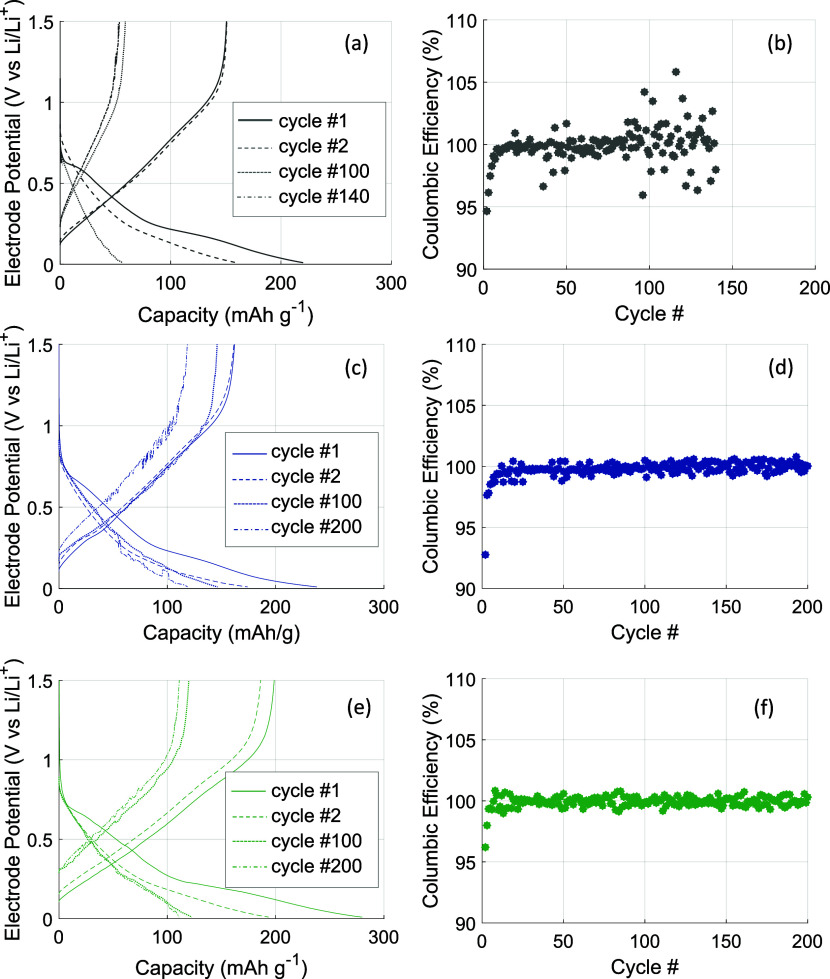
Voltage profiles and Coulombic efficiencies for structural electrodes with different matrix compositions: (a,b) DMMP39, (c,d) PC39, and (e,f) PCVC39.

The DMMP39-based SE formulation is not a suitable candidate for structural batteries, as the long-term behavior shows. All samples show a drastic decrease in charge and discharge capacity after around 30 cycles (Figures 1 andS2) with an average capacity retention of 29% after 140 cycles (Table S1). This effect is likely due to electrolyte decomposition and side reactions induced by DMMP, as suggested by the Coulombic efficiency data (Figure 2b) and the change in color of the separator (Figure S5, top). The high scatter in the Coulombic efficiency data (Figure 2b) and the subsequent cell failure further support the presence of side reactions.

The PCVC39-based structural electrode showed reduced capacity retention compared to the corresponding SE compositions without an additive. The data (Figures 1 and S2–S4, Table S1) show that 1 wt % of VC addition reduced the average capacity retention to 64% after 200 cycles (see Table S1), and this composition does not appear to be a suitable candidate for structural batteries. VC is commonly used as an additive to stabilize the SEI layer and prevent its degradation.^25^ One reason for the performance decrease could be that VC participates in the polymerization prior to cycling and thus cannot act in the desired manner.^26^

The voltage profiles for the previous SE compositions are listed in Figure 2. The 100th cycles of the different structural electrodes often show a noisier voltage profile. This behavior can indicate an issue with contact resistance between the current collector and the electrode, which is supported by the images of the PC39 electrode (Figure S5b) and could explain voltage losses. Voltage losses arising from electrical contact resistance can be as high as 20%^27^ and were found to be an issue in previous work.^9^

Structural electrodes with various SE compositions were reused from previous work.^9^ The samples were exposed to extended cycling and then electrochemically evaluated after a total of 100 cycles. For these samples, current collectors were adhered on both sides of the electrodes to reduce contact resistance (Figure S6). We found that the capacity retention was above 90% after 100 cycles for two different matrix compositions: the Thiol50 sample with thiol and the PC503M sample with increased salt concentration (see Figure 3). The improved capacity retention at higher salt concentrations, as also observed in our previous work, is attributed to the strong coordination of solvent molecules with Li ions, which reduces their susceptibility to side reactions.^28^ The slightly lower capacity retention for the PC50 sample, which only has an increased electrolyte content, also suggests a dependence on the SE composition (see Figure 3). Overall, increased liquid electrolyte content indicates to enhance capacity retention by reducing electrolyte degradation and improving interfacial stability, thereby minimizing active Li-ion loss. Increased electrolyte content may also compensate for decomposition losses caused by reactions between the electrolyte and freshly exposed electrode surfaces, which result from CF dimensional changes during cycling. The impedance data for these structural electrodes show that the most significant increase in internal resistance occurs after the first few cycles, with subsequent cycles contributing little to further changes in resistance (Figure S7).

**Figure 3:**
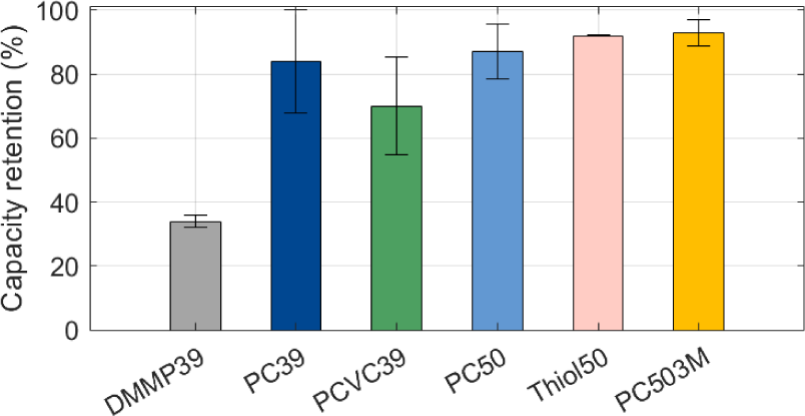
Capacity retention for structural electrodes with different SE compositions after 100 cycles.

Conclusively, the choice of solvent (i.e., porogen) and the way of adhering current collection seems to be important when it comes to the long-term performance of structural CF electrodes. The data also indicated that if additives are used, they should be injected after curing as they might react during the curing process.

### Effect of Long-Term Cycling on Morphology and Interfaces

The DMMP39 and PC39-based structural electrodes were further evaluated with respect to their interface morphology before and after cycling, as previous findings indicated significant electrochemical performance differences.^8,9^ Cross-sectional electron micrographs of uncycled and cycled DMMP39 (Figure 4) and PC39 (Figure 5) structural electrodes indicate differences in the interface morphology before and after cycling. The most noticeable difference in interface morphology induced by cycling appears to occur in the DMMP39-based structural electrodes. For these samples, galvanostatic cycling introduces or enlarges debonding gaps between fibers and the matrix (see Figure 4). Thus, for the DMMP39 sample, the micrographs suggest that the debonding is mainly governed by the expansion in the lithiation process.^13,14^ This effect seems less pronounced for the PC39-based system, as more debonding gaps seem to be present even before cycling (Figure 5). The FIB-SEM images for PC39 rather indicate the enlargement of gaps caused by cycling (Figure 5). A possible explanation for the debonding observed prior to cycling in the PC39 sample could be due to the presence of PC, which may induce a stronger interface debonding effect compared to DMMP. A previous study has found that moisture content indeed decreases mechanical properties by fiber–matrix interface debonding.^29^ Interestingly, the images of cycled PC39-based structural electrodes (see Figures 5 c,d andS8) show debonding gaps that closely match the formation predicted by a developed model,^30^ which shows the patterns to strain concentrations caused by Li-ion insertion in a negative structural battery electrode.

**Figure 4:**
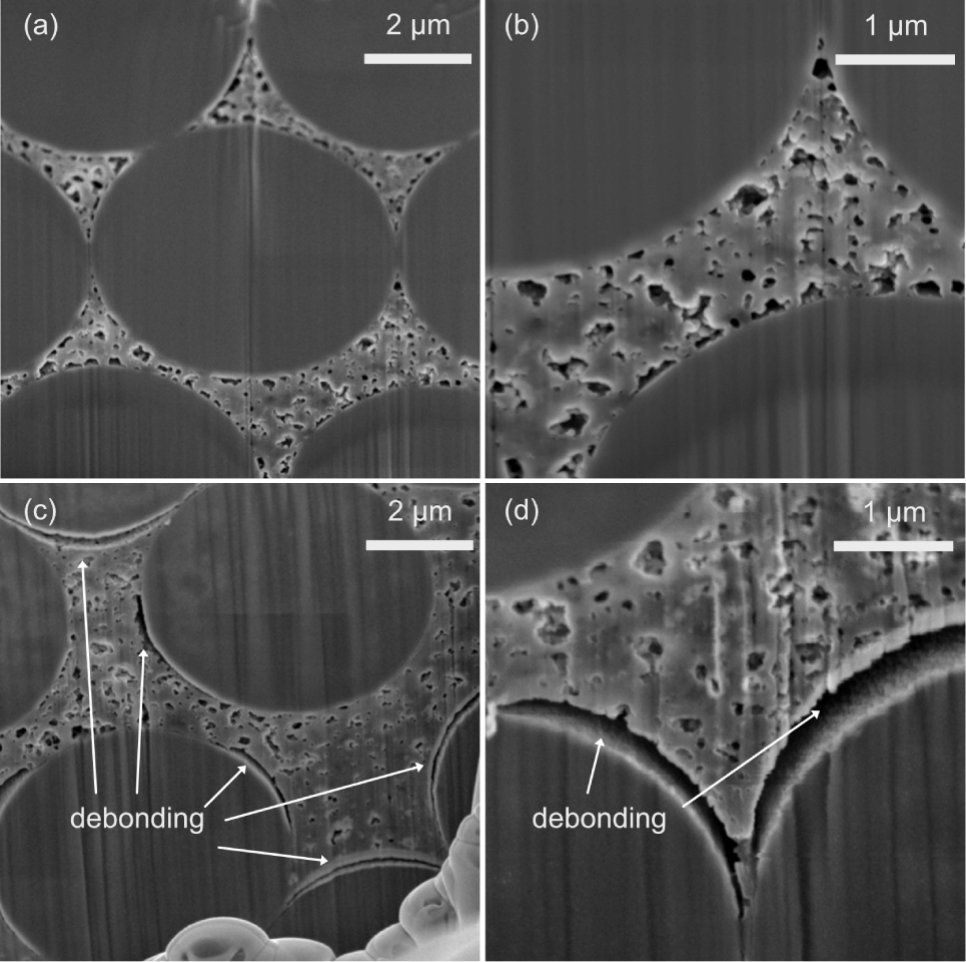
Electron micrographs of negative structural electrode cross sections with CFs embedded in a DMMP39-based SE at different magnifications of (a,b) an uncycled electrode and (c,d) a cycled electrode.

**Figure 5:**
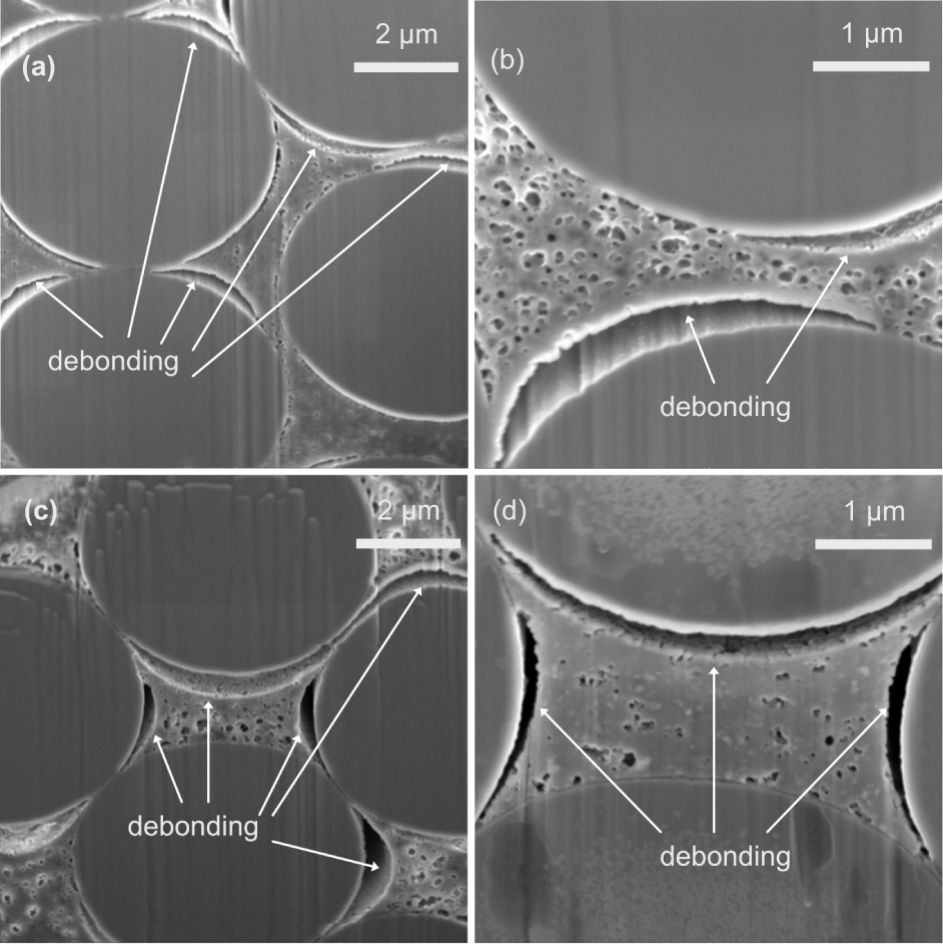
Electron micrographs of negative structural electrode cross sections with CFs embedded in a PC39-based SE at different magnifications of (a,b) an uncycled electrode and (c,d) a cycled electrode.

The SAXS data of cycled vs uncycled dried structural electrode cross sections support the previous finding that cycling has a larger impact on the DMMP39-based structural electrode (Figure 6). The data show large scattering intensity differences over the accessible *Q*-range between uncycled and cycled data of the DMMP39-based structural electrodes. The increase in scattering intensity is likely associated with the emerging delamination between the fibers and matrix related to Li-ion insertion. These emerging gaps will then introduce more scattering centers and increase the scattering intensity, as indicated in Figure 6. The scattering patterns show an average of summed images that were scaled to absolute intensities and normalized to thickness. Thus, a comparison of absolute intensities should be valid. For PC39, an increase in the intensity at the higher *Q*-range is observed. This is qualitatively in line with Figure 5, where small voids are visible. For a thorough size evaluation of the debonding gaps using the SAXS data, the sample-to-detector distance has to be increased as the debonding gaps appear to be larger than 100 nm in size (see Figures 4 and 5). We note that the measurement was constrained by the capabilities of the beamline facility as the sample–detector distance was already at the maximum.

**Figure 6:**
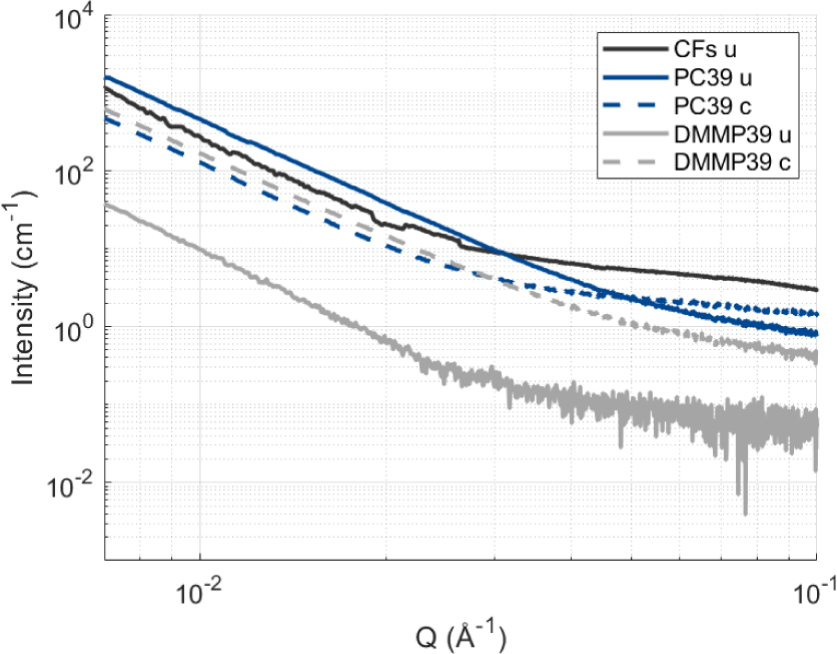
SAXS data showing *Q* vs normalized intensity using fiber length for different structural electrodes measured along the fiber direction. The u refers to uncycled structural electrodes, while c refers to cycled structural electrodes.

The curvature of the debonded areas also appears to be different between the DMMP39 and the PC39 samples (see Figures 4,5 and S8). The curvature of the debonded areas seems more uniform for the DMMP39 sample, which might relate to changes in the SE compositions, showing differences in morphology and mechanical properties. Another possibility is that the drying process affects both SE compositions in a different manner. The effect of drying conducted prior to SEM analysis is discussed in the subsequent section.

However, both types of electrodes (i.e., PC39 and DMMP39) show regions of adhesion and debonding between the polymer matrix and fibers even after long-term cycling (see Figures 4,5 andS8). The corresponding cryo-SEM micrographs for both uncycled and cycled PC39 structural electrodes also show intact CF–matrix interfaces (Figure S9 and S10). For a structural battery, contact with both the solid and liquid parts of the bicontinuous polymer–liquid electrolytes is essential to achieve both good energy density and high mechanical properties. Mechanically, the adhesion between the polymer matrix and the fibers is fundamental to a composite’s load-bearing property, as the matrix transfers loads between fibers. The partial debonding between the polymer and fibers in some areas therefore indicates that cycling likely reduces the mechanical performance of a unidirectional structural battery in the transverse direction, as indicated in previous work.^16^ Electrochemically, the Li ions need to intercalate into the CF structure, which requires access to the fibers through contact with the liquid part of the electrolyte. Thus, the partial debonding of the polymer might lead to increased rate performance because access to the fibers is enhanced. However, the detachment between the polymer and fibers can also reduce long-term stability due to the continued exposure of fresh fiber surfaces to the liquid electrolyte, which consumes active Li ions.

Thiol50-based structural electrodes were introduced to study whether the adhesive properties between the CFs and the polymer part of the matrix could be increased. Thiols are known to enhance interfacial adhesion through their ability to form covalent bonds with a variety of functional groups, thereby strengthening the fiber–matrix interface. Additionally, they can improve wetting and overall interfacial compatibility.^31,32^ The Thiol50 sample, however, showed an inhomogeneous distribution of bicontinuous electrolyte with insufficient impregnation of all fibers (Figure S11). Both cryo- and FIB-SEM images also showed the same kind of fiber–matrix debonding effects in the samples (see Figures S11 andS12). Thus, the addition of thiol does not seem to improve the interfacial adhesion in the desired manner and appears to alter the manufacturability.

In short, the analysis of the morphology and interfaces shows that the CF–matrix interface formation is dependent on SE composition. From a mechanical point of view, all investigated SE compositions will need to be optimized, as they show partial debonding between fibers and matrix even before cycling.

### Effect of Long-Term Cycling on Mechanical Properties

The evaluation of the mechanical properties before and after cycling is challenging due to sample sizes that are constrained by the manufacturing capabilities in the glovebox. As a result, the transverse properties could not be measured in a reliable manner, as the samples are too thin and fragile. However, the transverse properties of a full cell based on a PC39 formulation were measured in previous work.^16^ The results indicated a decrease in the transverse mechanical properties of the full-cell structural battery after cycling, identifying the negative electrode as the cause of the problem. The micrographs of the PC39-based structural electrodes also support these findings since the cycling appears to enlarge the fiber–matrix debonding, which likely explains the decrease in transverse properties.

An unconventional sample size was used to evaluate the longitudinal moduli of the structural electrodes. However, the method is sufficient for a qualitative comparison between cycled and uncycled samples for structural electrodes with different SE compositions. The unconventional sample size was inevitable due to the structural electrode manufacturing constraints of the glovebox. We included the “expected” modulus as a reference since there is a variation in the sample thickness, which introduces a relatively large scatter (see Figure 7 and S13–S18, Table S19). A structural electrode with a reduced porogen content (see Table 1, PC10) was also included as a reference for the mechanical properties. This composition should provide good load transfer between fibers since the matrix predominantly contains polymer.

**Figure 7:**
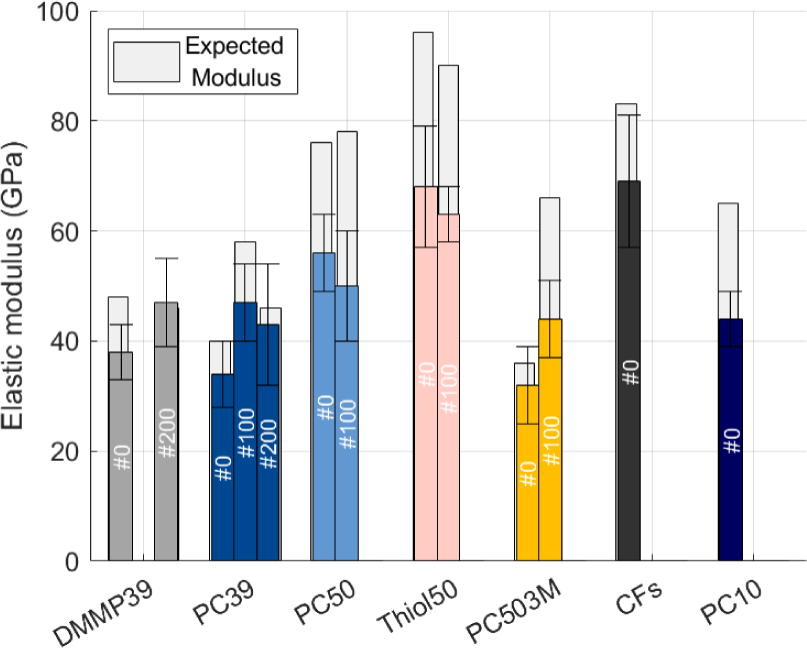
Elastic modulus comparison between structural electrodes with different SE formulations. The numbers on the respective bars indicate the number of cycles that the specific sample has performed. The expected modulus was calculated based on fiber volume fraction.

Figure 4 shows that long-term cycling has no significant impact on the longitudinal modulus, independent of the SE composition, which is in accordance with previous work.^16,33^ The modulus in the fiber direction is dominated by the CFs and thus these results indicate that the CFs are not significantly mechanically impaired by long-term cycling at low C-rates. The results also indicate that the Thiol50 sample generally shows a higher modulus than other matrix compositions, which is associated with the sample thicknesses. Different matrix compositions can result in different sample thicknesses even when using the same manufacturing technique, which is due to differences in resin viscosities. It is notable that the Thiol50 formulation produces much thinner samples and thus results in a generally higher modulus. The decreased sample thickness is most likely due to the resin not fully penetrating the CF tow, as indicated in the previous section (see Figure S11). This effect can be related to different reaction rates.^9^ The deviation from the expected modulus is generally found in the sample preparation and testing method, which was designed for unusually small sample sizes. In terms of sample preparation, thickness can vary within a specimen (see Figure S20) and the hand-manufactured, cut, and tabbed samples can lead to misalignments of the CFs.

### Effect of Drying on the Bicontinuous Electrolyte

The previous section discussed FIB- and cryo-SEM images, which are based on structural electrodes in their “dry” (i.e., without liquid electrolyte) and “wet” (i.e., with liquid electrolyte) states, respectively. The effect of liquid extraction and drying was evaluated on the SE morphology without CFs. The results show that drying seems to alter the morphology of the bicontinuous electrolytes, as shown in Figure 8, and the SAXS data are plotted in Figure 9. The drying process shrinks the SE films to different extents, depending on the SE formulation, where the Thiol50 sample seems to shrink the most (see Figure 8). In addition, the opacity seems to increase after drying for all SE compositions, indicating that drying leads to the collapse of smaller pores (see Figure 8). These findings correlate with previous work on porous polymers, which found a dependency on the degree of shrinkage upon drying that is related to pore sizes, where smaller pores collapse more easily.^34,35^

**Figure 8:**
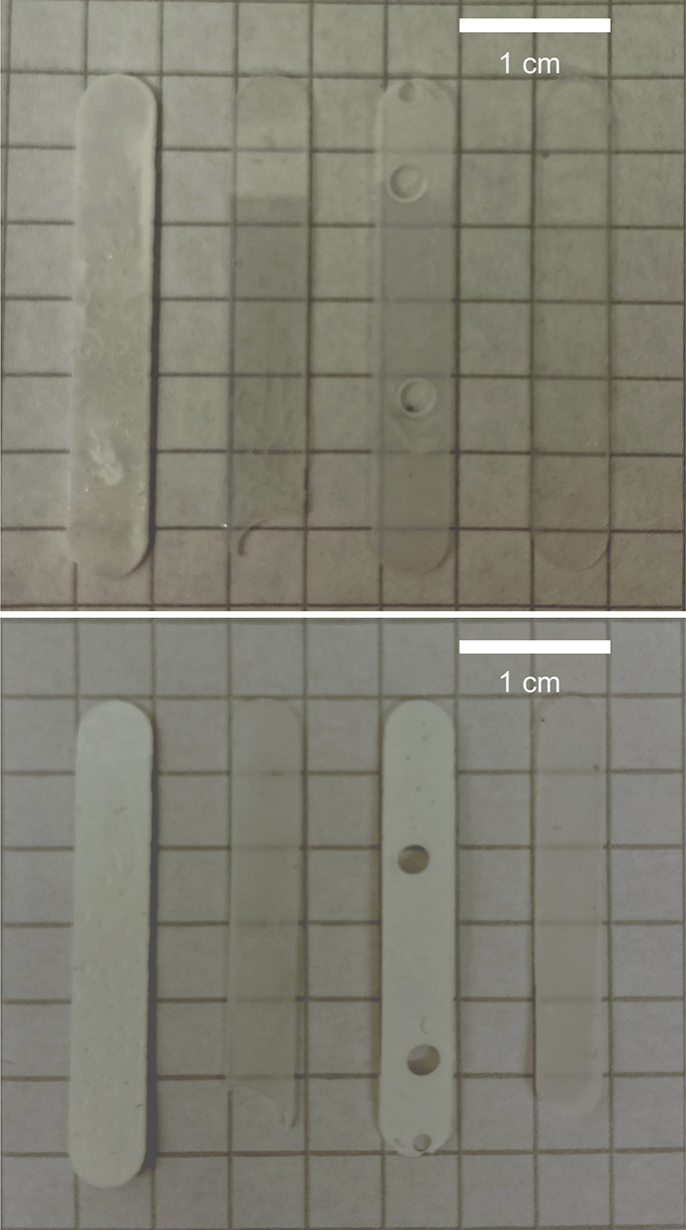
Pictures of SE films before (top) and after (bottom) drying. Different SE formulations from left to right: DMMP39, PC39, PC50, and Thiol50.

**Figure 9:**
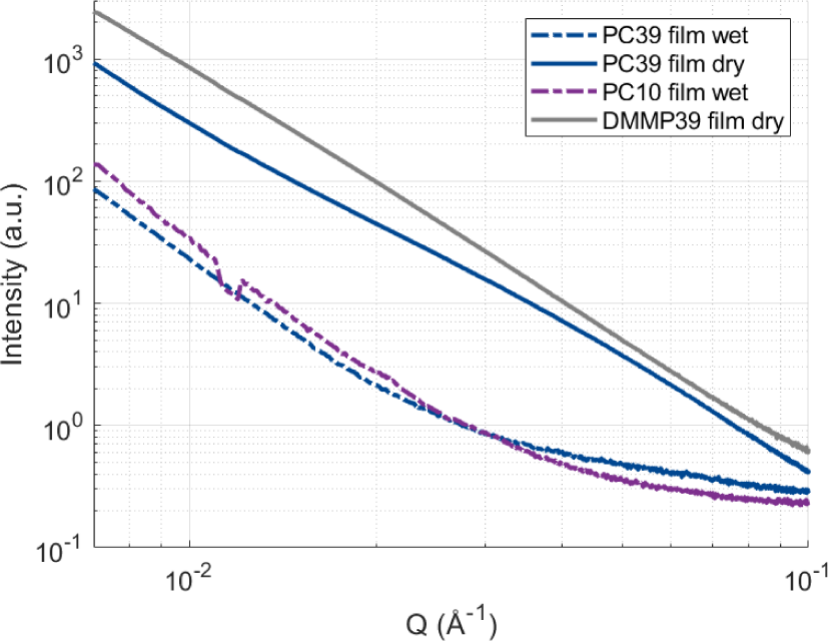
SAXS image of free-standing SE films: wet (PC39), dry (PC39, DMMP39), and containing 10% liquid (PC10). The kink around 1.4 × 10^–2^ Å for PC10 stems from missing intensity due to a detector gap.

The SAXS data show a similar trend with an increase in scattering intensity for the dried samples (PC39 and DMMP39). Furthermore, the wet sample lines up more with the PC10 sample, which is also transparent and should indicate small to no pores due to the low liquid content. When fitting these curves using the power law and assuming spherical pores/voids (see Figure S21), the data also indicate a shift in average pore diameter from 6.2 ± 1.2 to 10.6 ± 3.2 nm when comparing wet and dry PC39 electrolyte samples (see Table S22), respectively. The films also appear to have pores larger than 100 nm, as the continuation of the slope in Figure 9 indicates. As a result, both the FIB-SEM and SAXS data also support previous work findings that indicated the formation of larger pores for the DMMP39 compared to the PC39 matrix composition.^9,36^ The data further suggest that drying seems to change from spherical shapes to more nonspherical pores as the power parameter shifts from 3.9 to 3 (see Table S22). The dry DMMP39 shows an average pore diameter of around 35.8 ± 21.5 nm and contains pore sizes above 100 nm. PC39 films (without CFs) were imaged in dry and cryo-states with frozen liquid; however, the difference in fracture surfaces of the samples makes the comparison difficult and needs a refinement in methodology (see Figure S23). Relating these SAXS results to the FIB-SEM images (see Figures 4 and[Fig fig5]) confirms the differences in pore sizes for the dried DMMP39 and PC39 samples and shows nonspherical voids in both dried samples. The difference in debonding morphologies between PC39 and DMMP39 (see Figures 4 and[Fig fig5]) may also be influenced by drying effects, as the PC39 formulation exhibits smaller pores and appears to shrink more upon drying, potentially causing greater morphological distortions at the interfaces.

## Conclusion

This study examined the long-term properties of CF-based structural electrodes with varying matrix compositions. Utilizing advanced characterization techniques, the CF–matrix interfaces and their ability to accommodate cyclic stresses induced by Li-ion insertion were analyzed. The findings indicate that matrix composition exerts a substantial influence on the electrochemical longevity of structural negative electrodes, with porogen structure playing a pivotal role. PC-based electrolytes without VC as an electrolyte additive showed the most promising electrochemical long-term properties. However, current collector adhesion indicates to be a critical factor, contributing to an increase in cell resistance and capacity fade. A higher liquid electrolyte content (50%) and salt concentration generally improved the capacity retention. Mechanically, the CF-composite modulus remained largely unaffected by long-term cycling at low current densities. FIB-SEM and SAXS proved to be effective for studying CF–matrix interface morphology at both local and global scales. Cryo-SEM is a viable and complementary technique to investigate samples retaining the liquid electrolyte and showed the ability to resolve the nanoporosity of the SE. The overall results show that electrochemical cycling induced fiber–matrix debonding with its extent dependent on matrix composition. However, the investigated SEs also exhibited adhesion between fibers and the polymer part of the SE after prolonged cycling—an essential feature for high-performance structural batteries. These findings provide key insights into optimizing matrix design for durable structural battery applications.

## Supplementary Material



## Notes

Detailed information on capacity retention and additional cycling data, electrochemical impedance data, FIB-SEM images, cryo-SEM, fitting parameters of SAXS curves, stress– strain curves, and values on mechanical properties (PDF)
