# Supplementary material for: Interface Evolution and Long-Term Performance of Negative Carbon Fiber Structural Electrodes
Source: ACS Omega. 2025 Jul 1;10(27):29109–18. doi: 10.1021/acsomega.5c01630 (PMC12268396; doi:10.1021/acsomega.5c01630)
Supplement: Supplementary file 1 [file ao5c01630_si_001.pdf]

Interface Evolution and Long-term Performance of Negative Carbon Fiber Structural Electrodes

Lynn M. Schneider[1], Benedikt Sochor[2,3], Marcus Johansen[4], Fang Liu[4], Göran Lindbergh[5], Dan Zenkert[1], Stephan Roth[2,6], Sarathlal Koyiloth Vayalil[2,7], Louise Lebret[1].

[1] Department of Engineering Mechanics, KTH Royal Institute of Technology, SE-100 44 Stockholm, Sweden.

[2] Deutsches Elektronen-Synchrotron DESY, Notkestr. 85, 22607 Hamburg, Germany.

[3] Advanced Light Source, Lawrence Berkeley National Laboratory, CA 94720 Berkeley, USA.

[4] Department of Industrial and Materials Science, Chalmers University of Technology, SE-412 96, Gothenburg, Sweden.

[5] Department of Chemical Engineering, KTH Royal Institute of Technology, SE-100 44 Stockholm, Sweden.

[6] Department of Fibre and Polymer Technology, KTH Royal Institute of Technology, SE-100 44 Stockholm, Sweden.

[7] Applied Science Cluster, UPES, Dehradun, Uttarakhand 248007, India.

Table 1: Cycling data, including lithiation and delithiation capacities for multiple structural half-cells with different SE formulations. The bold numbers are averages of the numbers below. The different terms (S1, S2, S3, S4) under each formulation DMMP39, PC39 and PCVC39 refer to replicates of that specific formulation.

	**cycle #1 lithiation capacity (mAh g^{-1})**	**cycle #1 delithiation capacity (mAh g^{-1})**	**cycle #1 capacity retention (%)**	**cycle #5 delithiation capacity (mAh g^{-1})**	**cycle #100 delithiation capacity (mAh g^{-1})**	**cycle #100 capacity retention (%)**	**cycle #140 delithiation capacity (mAh g^{-1})**	**cycle #140 capacity retention (%)**	**cycle #200 delithiation capacity (mAh g^{-1})**	**cycle #200 capacity retention (%)**
**DMMP39**	**-**	-	-	**155**	**54**	**34**	**46**	**29**	**-**	-
S1	220	151	69	164	59	36	54	33	-	-
S2	-	-	-	152	54	35	42	28	-	-
S3	-	-	-	149	48	32	41	27	34	23
**PC39**	**223**	**169**	**75**	**171**	**144**	**86**	**139**	**84**	**109**	**79**
S1	238	162	68	141	146	103	128	91	118	84
S2	232	212	92	206	120	58	104	51	94	46
S3	198	132	66	106	98	92	109	103	115	108
S4	-	-	-	231	212	92	212	92	-	-
**PCVC39**	**221**	**137**	**58**	**143**	**104**	**70**	**99**	**66**	**96**	**64**
S1	270	199	74	156	120	77	120	77	111	71
S2	173	75	43	94	49	52	46	49	46	49
S3	218	-	-	180	144	80	131	73	131	73

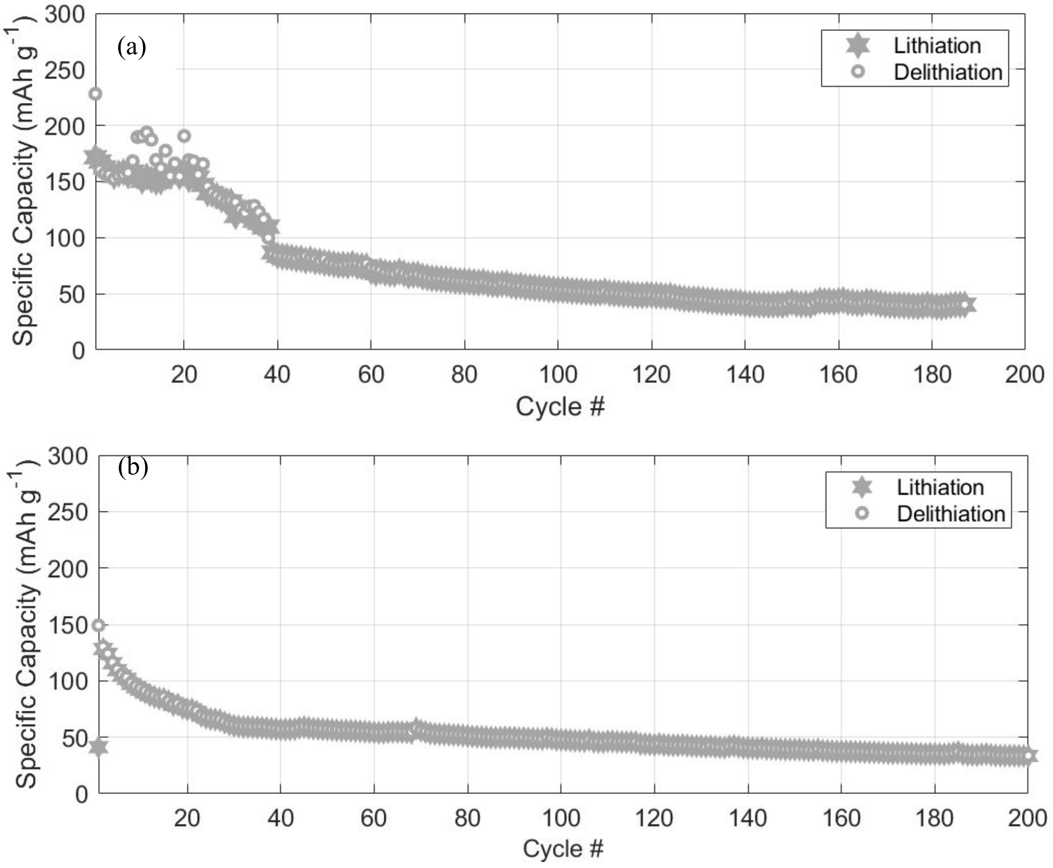

Figure S2: Cycle vs capacity curve for DMMP39-based structural electrodes a) S1, b) S3. S2 is presented in the main publication.

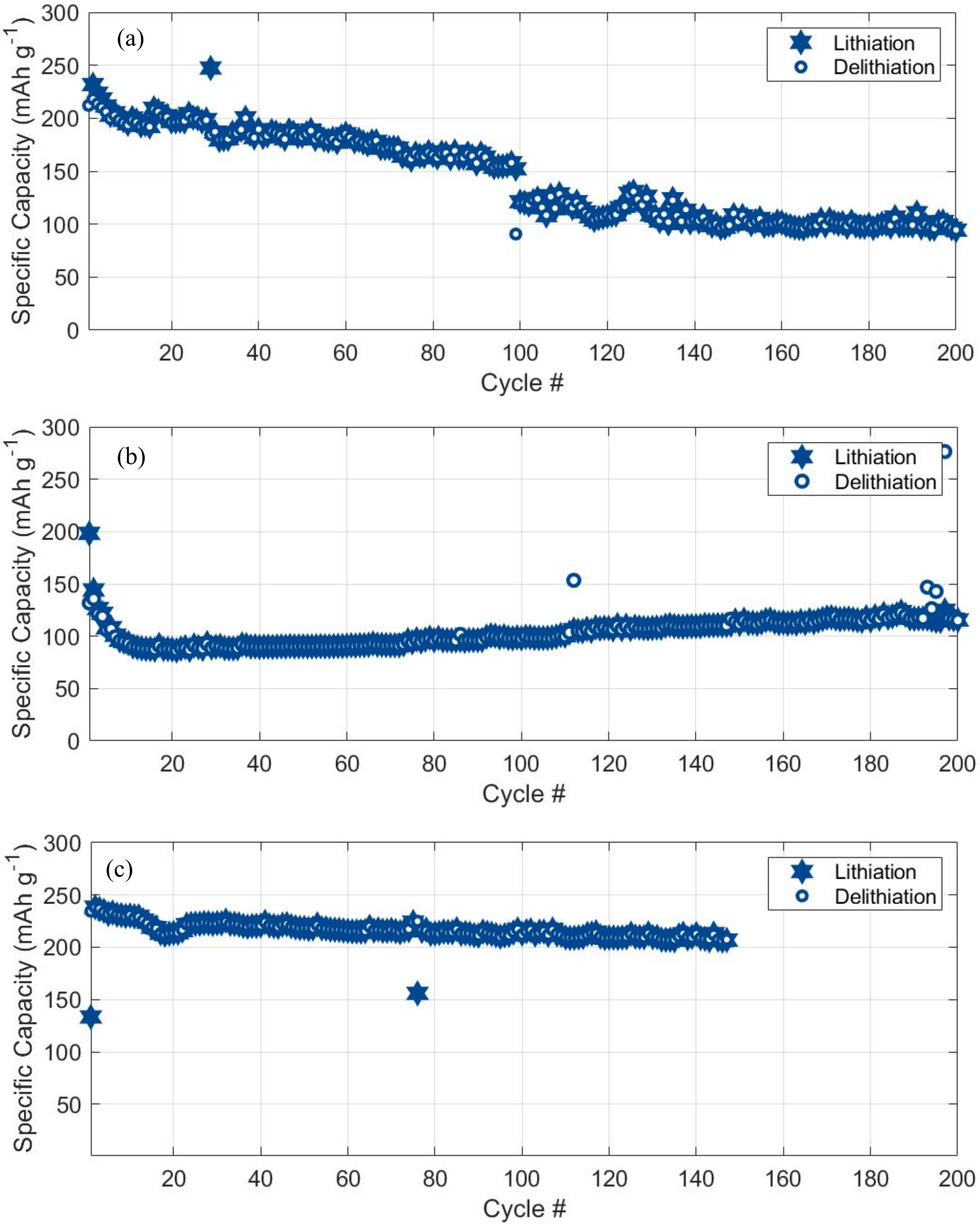

Figure S3: Cycle vs capacity curve for a PC39-based structural electrodes a) S2, b) S3 and d) S4. S1 is presented in the main publication.

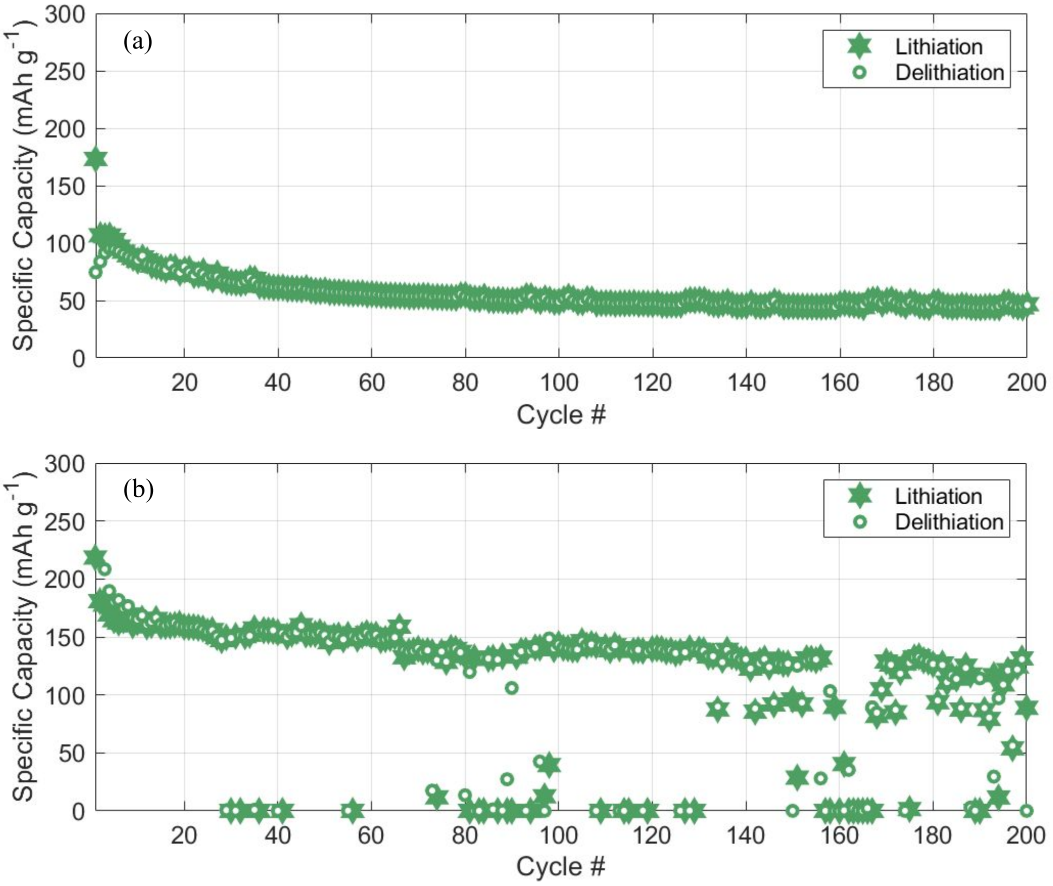

Figure S4: Cycle vs capacity curve for PCVC39-based structural electrodes a) S2, and b) S3. S1 is presented in the main publication. Note that some data points in S3 showing 0 specific capacity were related to issues with the used potentiostat.

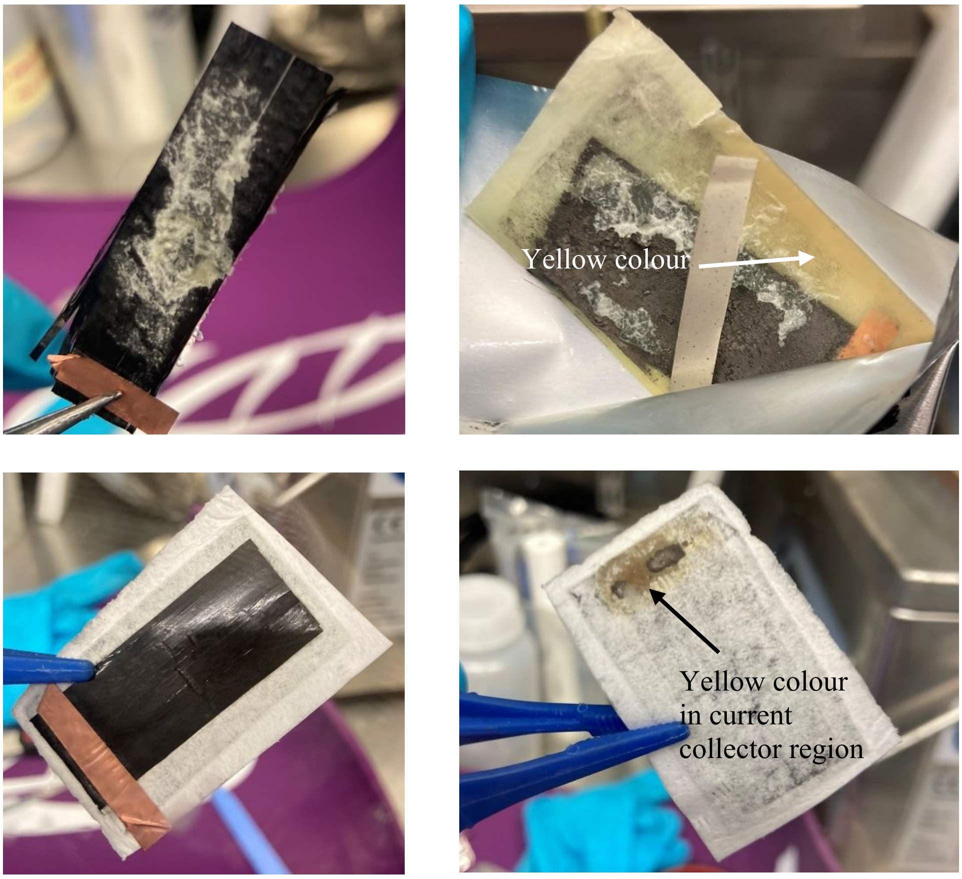

Figure S5: pictures of structural electrodes after cycle #200 DMMP39 (top) PC39 (bottom).

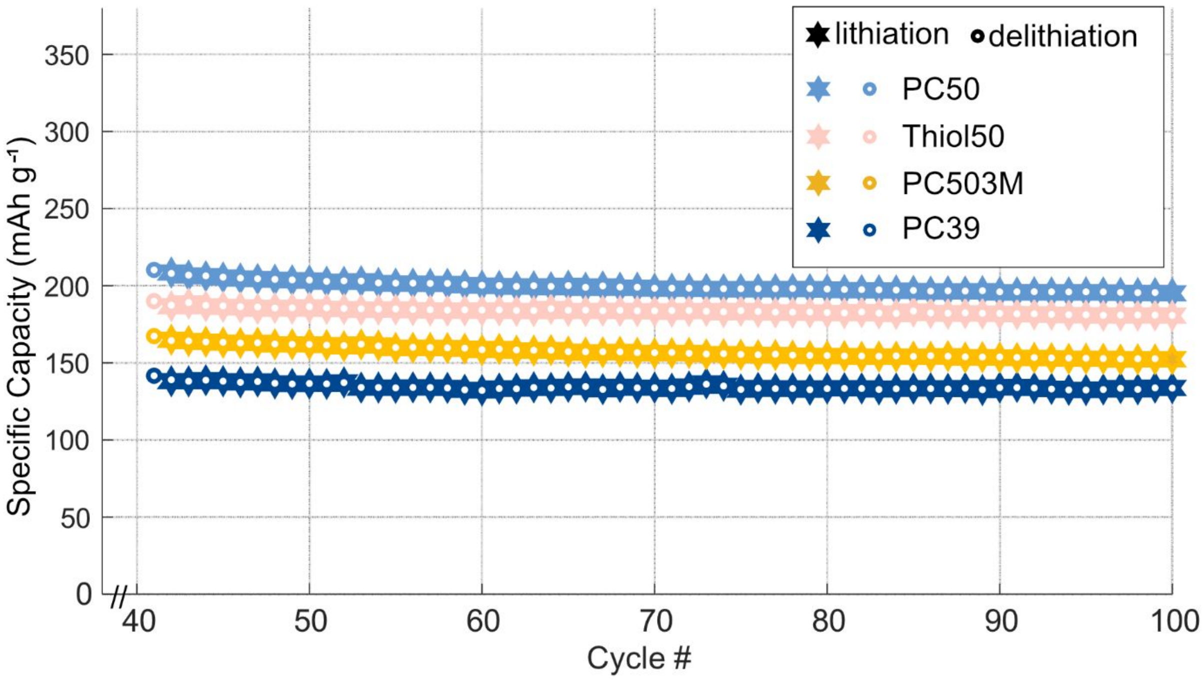

Figure S6: Capacity vs cycle number (lithiation x and delithiation o) one current density of 18.6 mAh g^{-1} from cycles 40 to 100 of CF-SE half-cells with different SE formulations (PC39, PC50, PC503M, Thiol50).

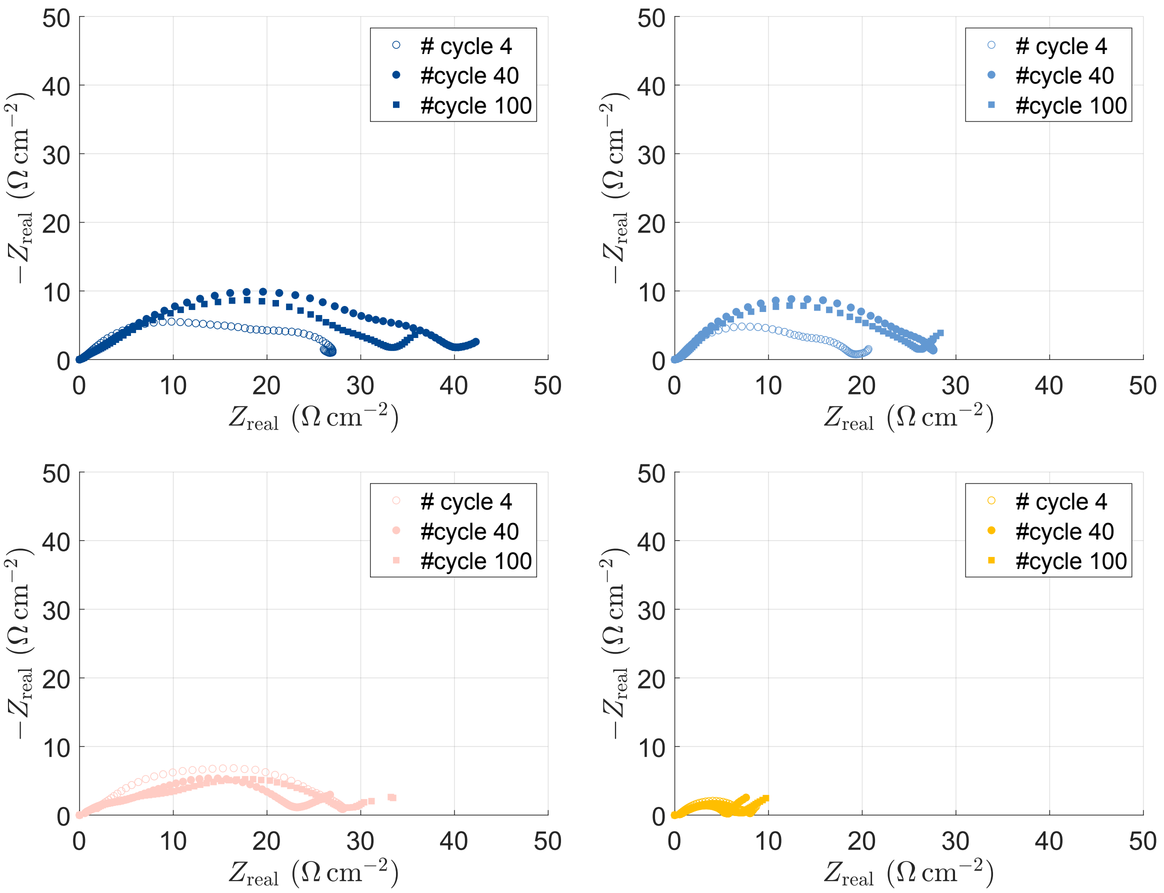

Figure S7: Impedance data (100kHz - 6mHz) after 4, 40 and 100 cycles for different structural electrodes combining carbon fibres and their respective SE formulation (a) PC39, (b) PC50 (c) Thiol50 (d) PC503M. Note that the impedance data for cycle #4 and #40 was reproduced from Schneider et al. under CC BY 4.0[3].

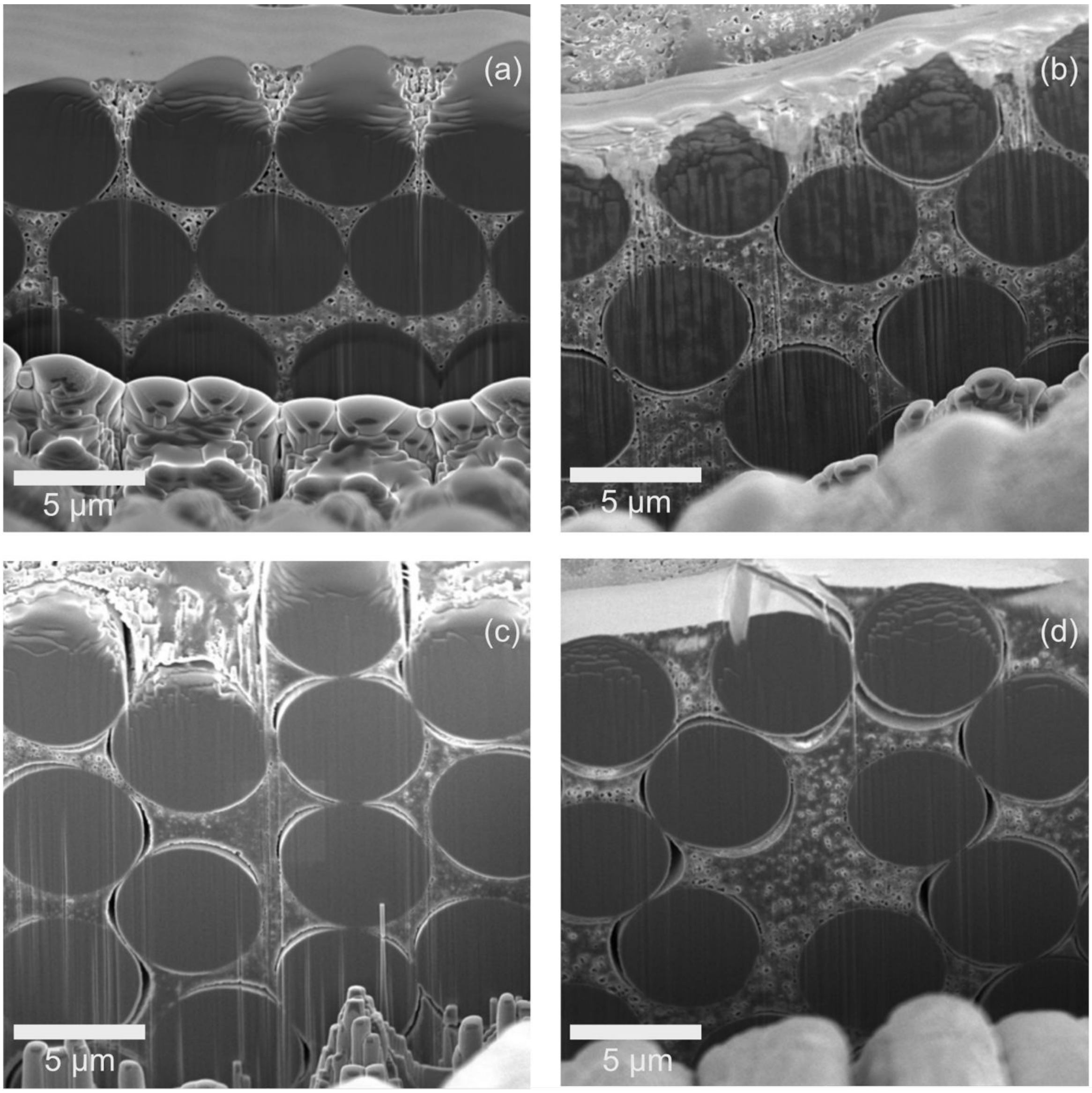

Figure S8: FIB-SEM image (a) uncycled and (b) cycled DMMP39, (c) uncycled and (d) cycled PC39 cross-sectional view of carbon fibres - bicontinuous matrix interfaces.

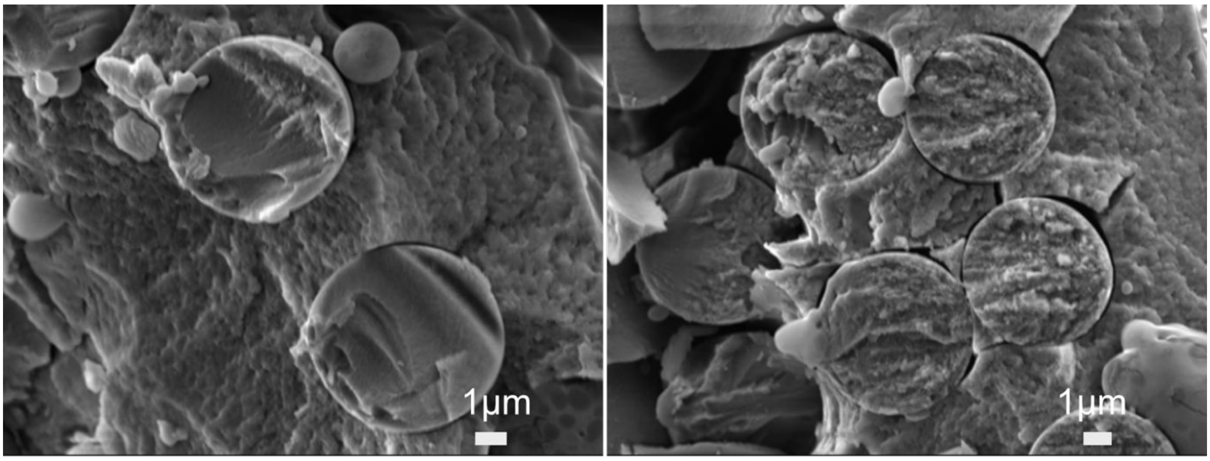

Figure S9: Cryo-SEM cross-section of an uncycled PC39 structural electrode.

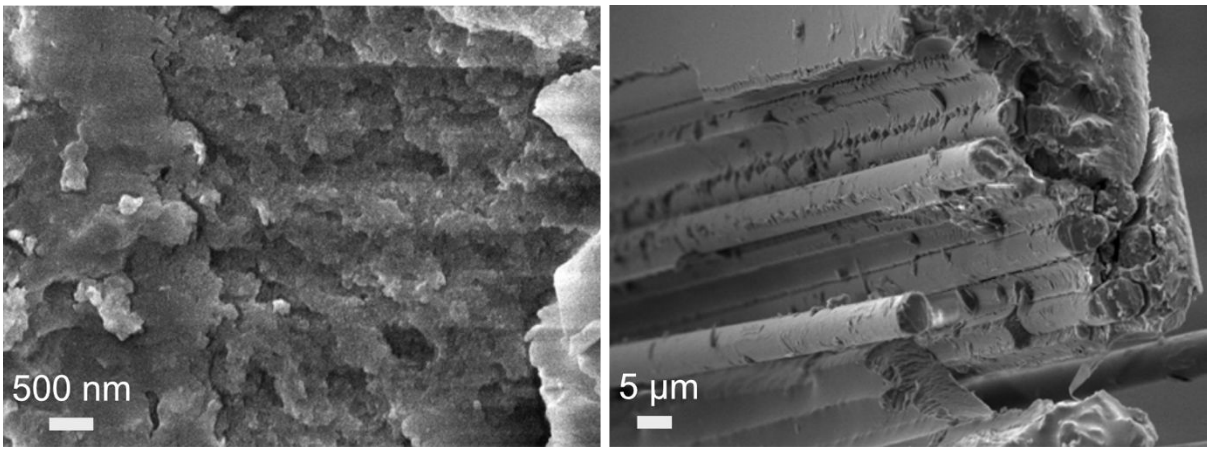

Figure S10: Cryo-SEM cross-section of a cycled PC39 structural electrode.

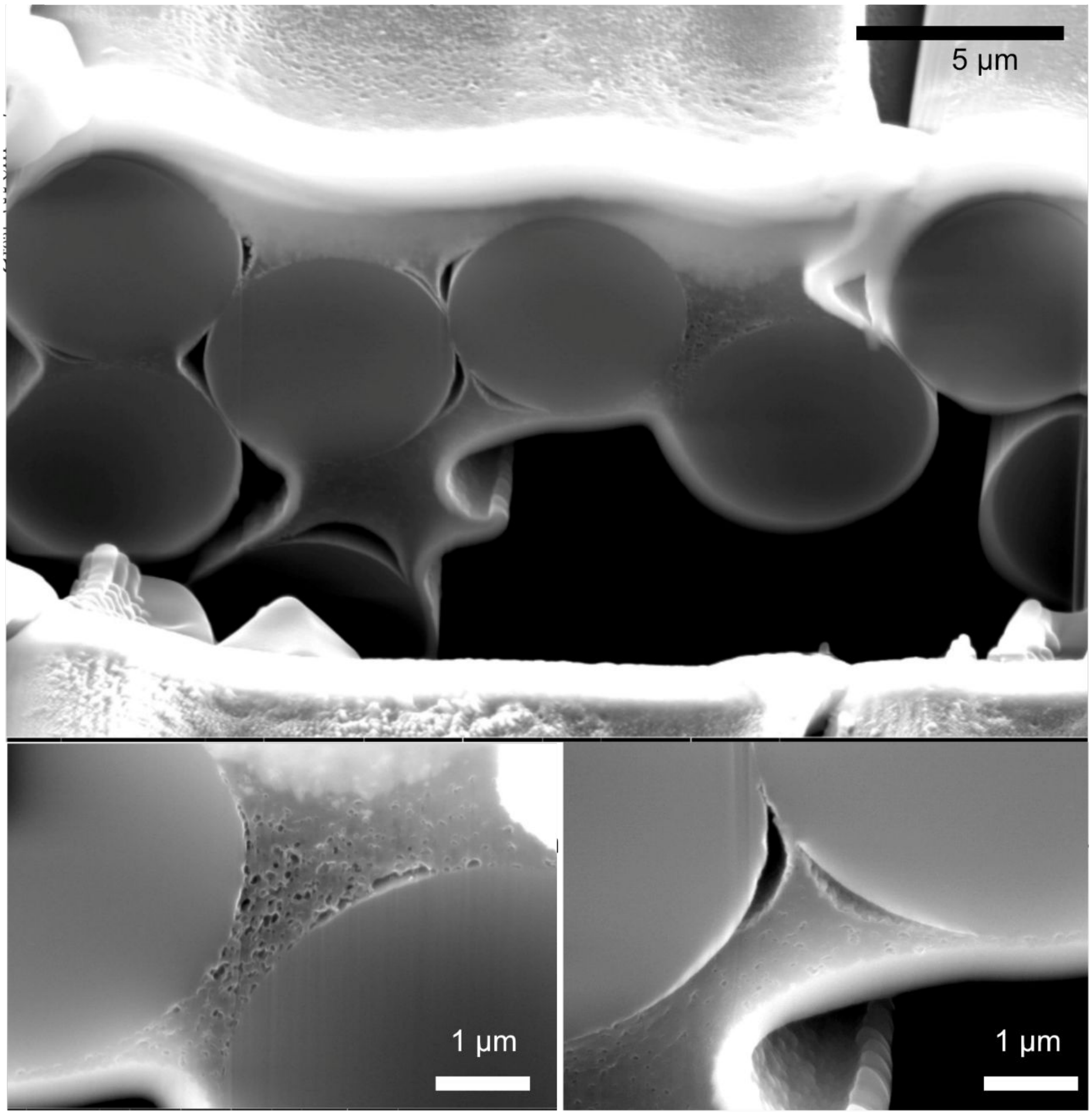

Figure S11: FIB-SEM image of a CF-matrix interface of an uncycled structural electrode based on Thiol50.

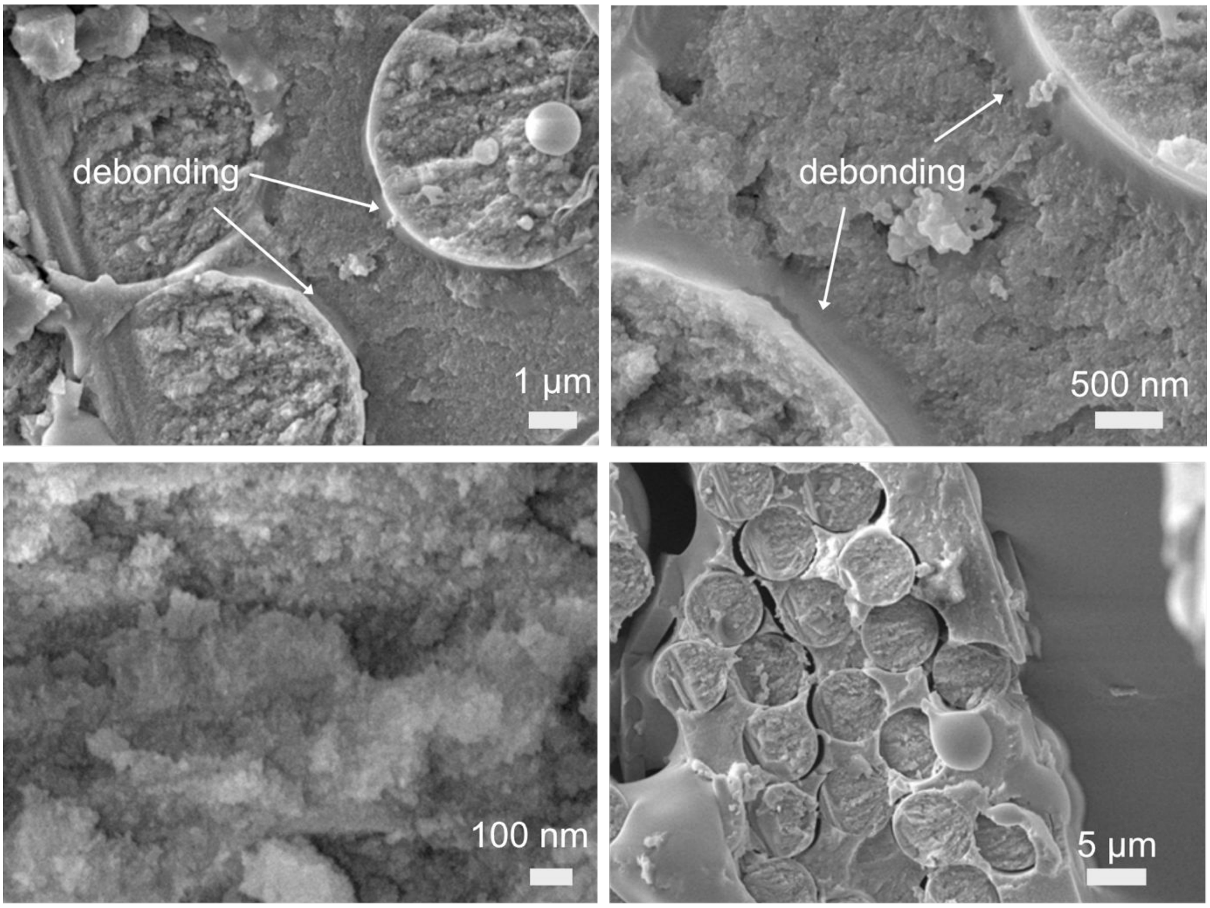

Figure S12: Cryo-SEM of a cycled structural electrode of Thiol50.

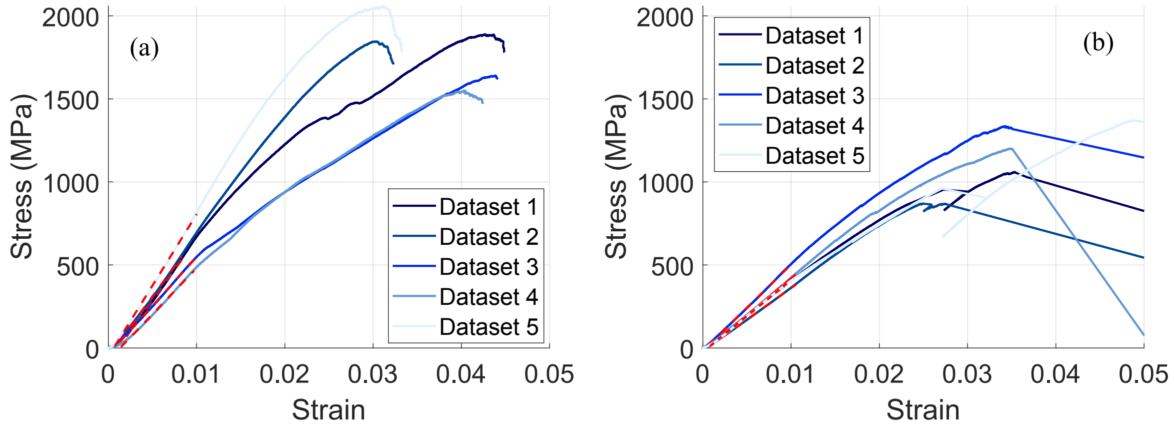

Figure S13: Stress strain curve of T800S CFs (a) uncycled without SE and (b) uncycled with SE (PC10). The blue lines indicate samples that failed, black lines indicate tested specimen without failure, red lines indicate linear approximations.

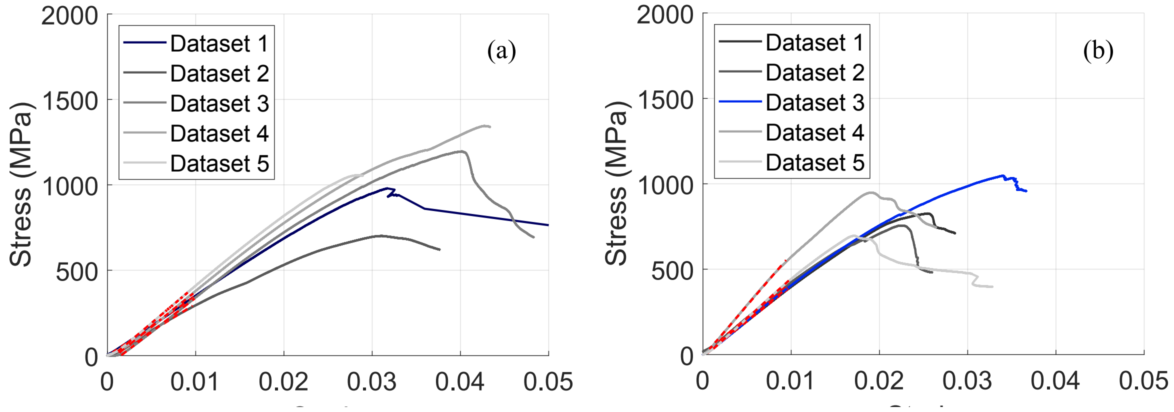

Figure S14: Stress strain curve of a structural electrode based on DMMP39 (a) uncycled and (b) cycled #140. The blue lines indicate samples that failed, black lines indicate tested specimen without failure, red lines indicate linear approximations.

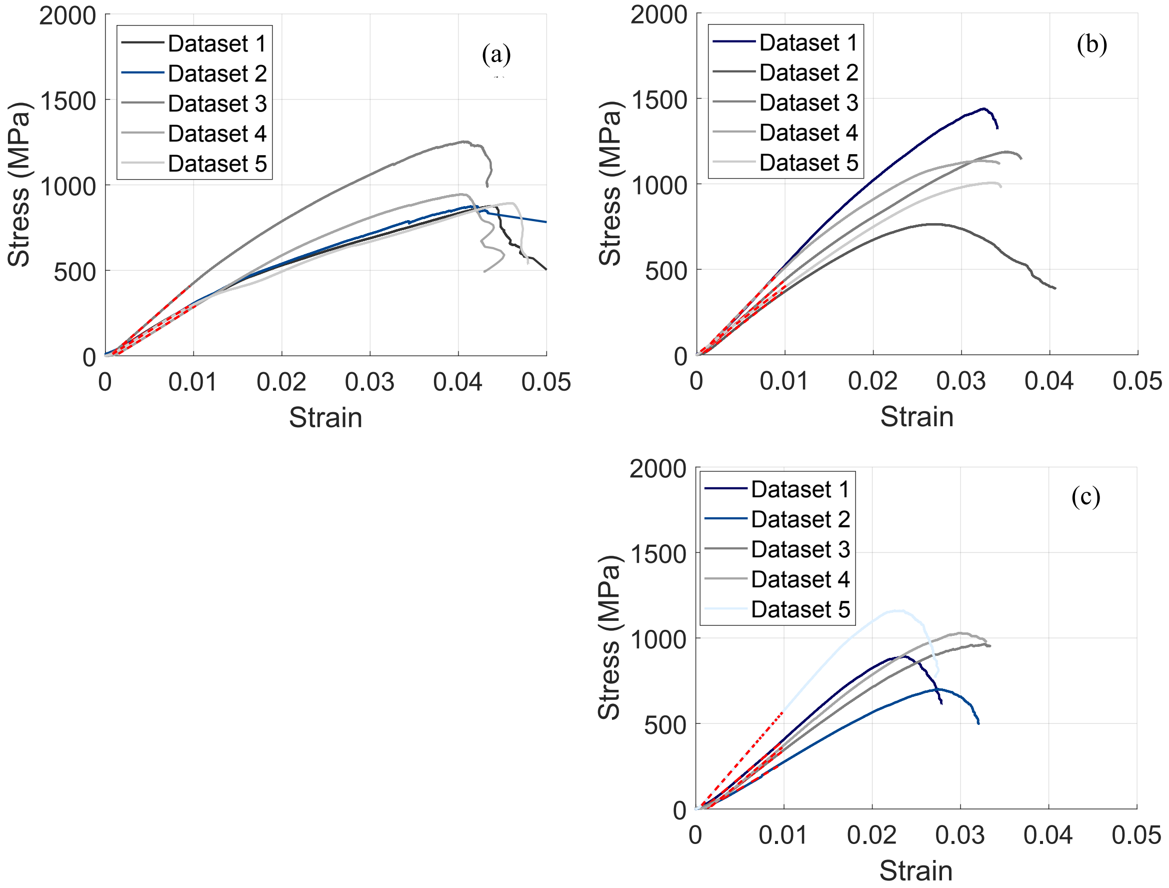

Figure S15: Stress strain curve of a structural electrode based on PC39 (a) uncycled and (b) cycled #100 (c) cycled #200. The blue lines indicate samples that failed, black lines indicate tested specimen without failure, red lines indicate linear approximations.

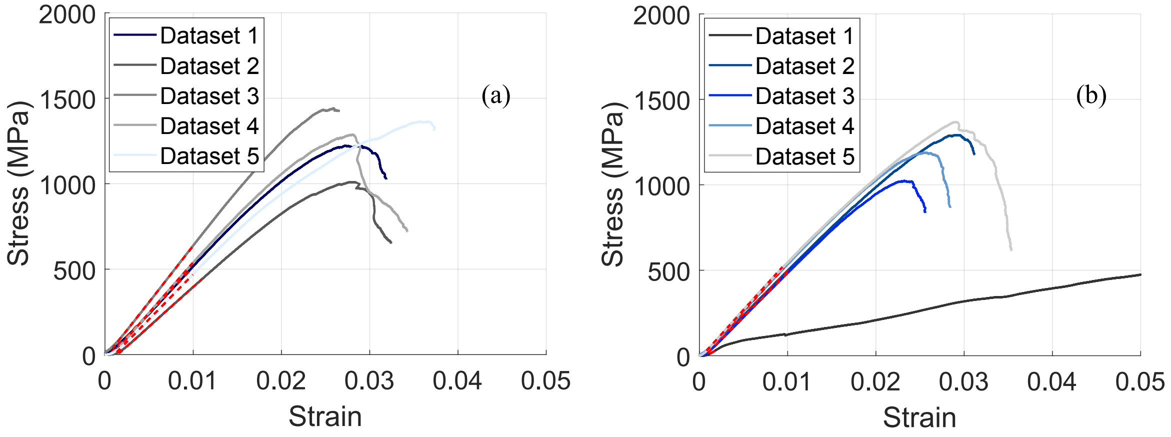

Figure S16: Stress strain curve of a structural electrode based on PC50 (a) uncycled and (b) cycled #100. The blue lines indicate samples that failed, black lines indicate tested specimen without failure, red lines indicate linear approximations.

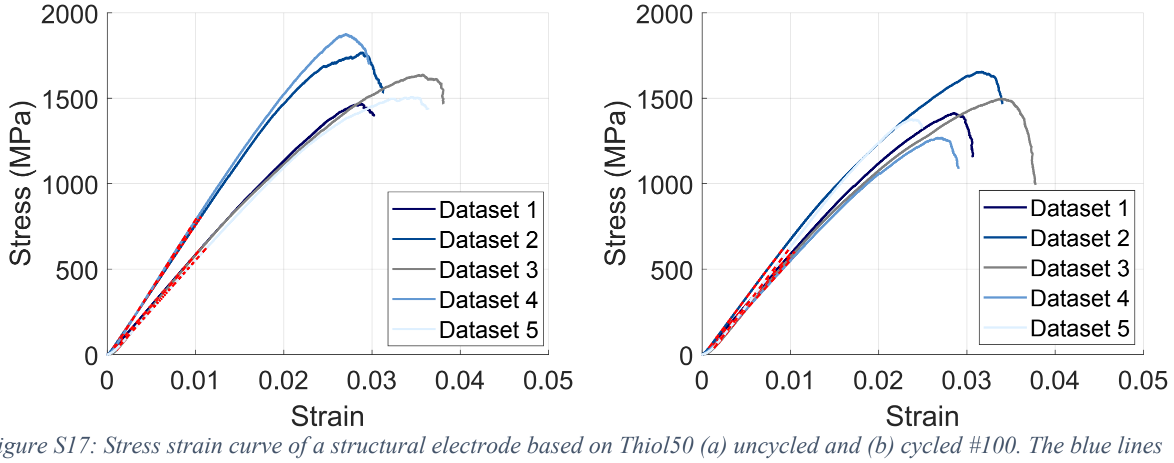

Figure S17: Stress strain curve of a structural electrode based on Thiol50 (a) uncycled and (b) cycled #100. The blue lines indicate samples that failed, black lines indicate tested specimen without failure, red lines indicate linear approximations.

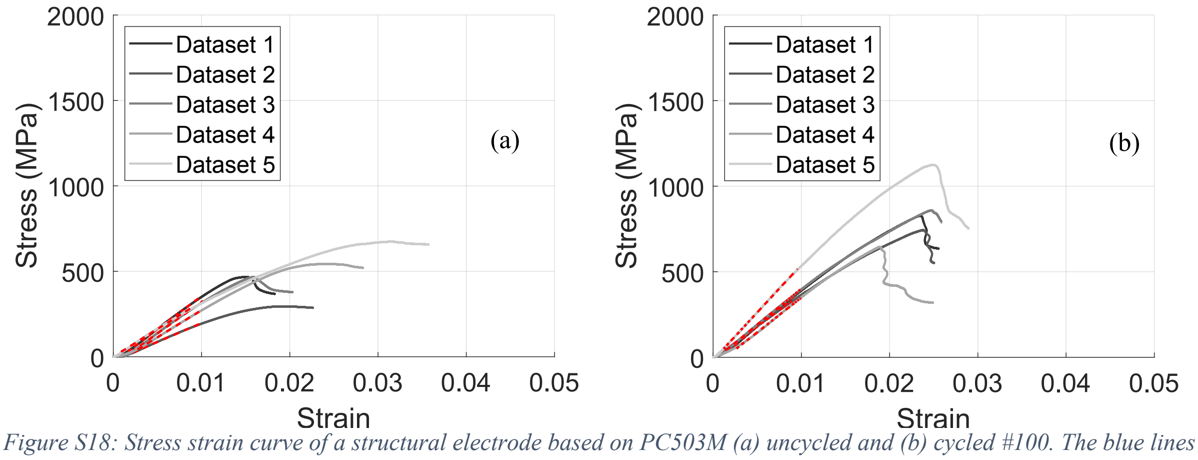

Figure S18: Stress strain curve of a structural electrode based on PC503M (a) uncycled and (b) cycled #100. The blue lines indicate samples that failed, black lines indicate tested specimen without failure, red lines indicate linear approximations.

Table S19: Elastic modulus in longitudinal direction before and after electrochemical cycling in GPa.

Sample	0 cycles	Expected modulus	100 cycles	Expected modulus	200 cycles	Expected modulus
**DMMP39**	38 ±5 GPa	48 GPa	-	-	47 ±8 GPa	46
**PC39**	34 ±6 GPa	40	47 ±7 GPa	58	43 ±11 GPa	46
**PC50**	56 ±7 GPa	76 GPa	50 ±10 GPa	78	-	-
**Thiol50**	68 ±11 GPa	96	63 ± 5 GPa	90	-	-
**PC503M**	32 ± 7 GPa	36 GPa	44 ±7 GPa	66	-	-
**T800s**	69 ± 12 GPa	83	-	-	-	-
**PC10**	44 ±5 GPa	65	-	-	-	-

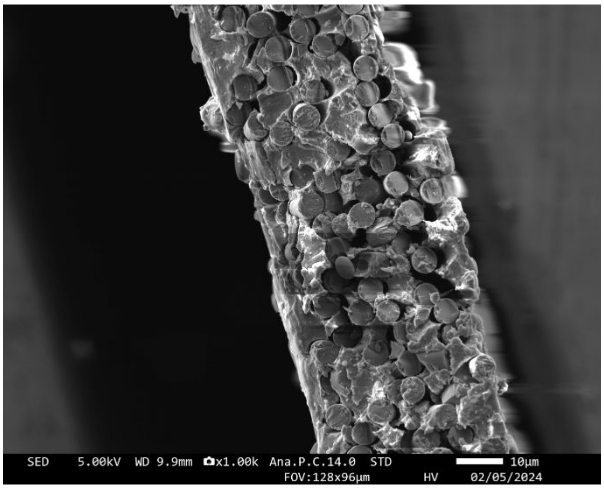

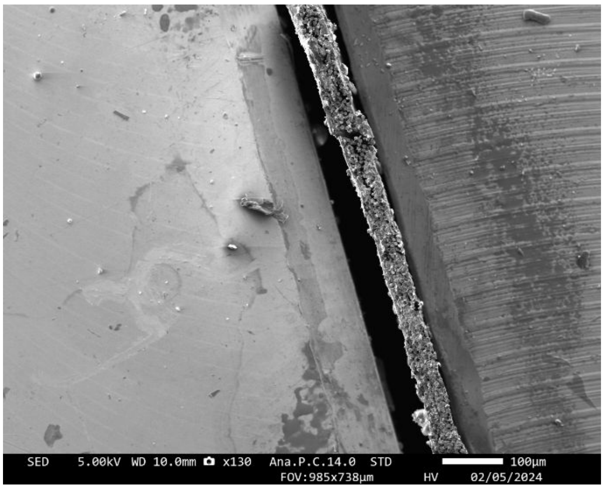

Figure S20: Cryo-SEM images of an uncycled structural electrode cross-section based on PC39.

For the SAXS data fitting, the measured intensities I were modelled using

$$I = C_1 \cdot q^{-p} + I_{sphere} + BG$$

with the scaling constant C_1, the scattering vector $q = \frac{4\pi}{\lambda} sin\theta$, where λ is the X-ray wavelength and θ half of the scattering angle, the Porod exponent p, the constant scattering background BG and the sphere contribution [1].

$$I_{sphere} = C_2 \cdot \int_{-PDI \cdot R_0}^{PDI \cdot R_0} \left(\frac{\sin q R_0 - q R_0 \cos q R_0}{(q R_0)^3} \right)^2 exp\left(-\frac{(R - R_0)^2}{2(PDI \cdot R_0)^2} \right) dR,$$

where C_2 is a scaling constant, PDI the polydispersity index and R_0 the mean sphere radius. For the modeling a Gaussian distribution of the sphere radii is assumed. For fitting the model to the experimental data, a simple Levenberg-Marquardt algorithm was used to optimize the least-square difference at each data point as it is routinely implemented in several python packages, e.g. scipy[2].

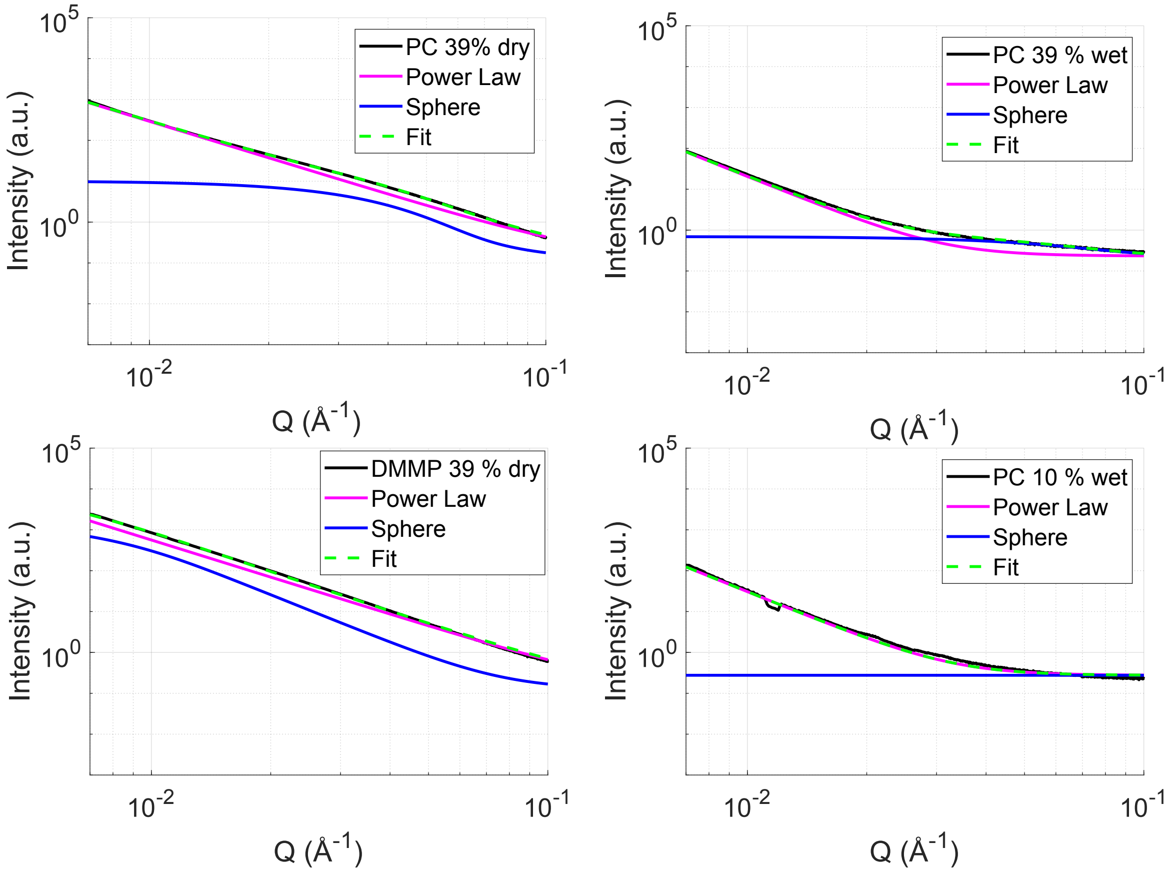

Figure S21: SAXS data fits for different structural electrolyte films.

Table S22: Fitting parameters and corresponding errors for different samples.

**Sample Names**	**C_1**	**p**	**C_2**	**R_0 (nm)**	**PDI**	**BG**
**DMMP39 dry**	5.2×10^{-4}	3.0	1.9×10^{-5}	17.9	0.6	0.1
	2.3×10^{-6}	1.3×10^{-3}	2.7×10^{-7}	0.1	0.01	2.8×10^{-3}
**PC39 wet**	3.1×10^{-7}	3.9	1.8×10^{-6}	3.1	0.2	0.2
	6.0×10^{-9}	4.0×10^{-3}	7.5×10^{-7}	0.6	0.2	2.1×10^{-3}
**PC39 dry**	3.3×10^{-4}	3.0	7.0×10^{-6}	5.3	0.3	0.1
	1.9×10^{-6}	1.2×10^{-3}	6.5×10^{-8}	0.02	0.005	2.0×10^{-3}
**PC10 wet**	4.2×10^{-7}	3.9				0.3
	5.5×10^{-9}	2.6×10^{-3}				7.4×10^{-4}

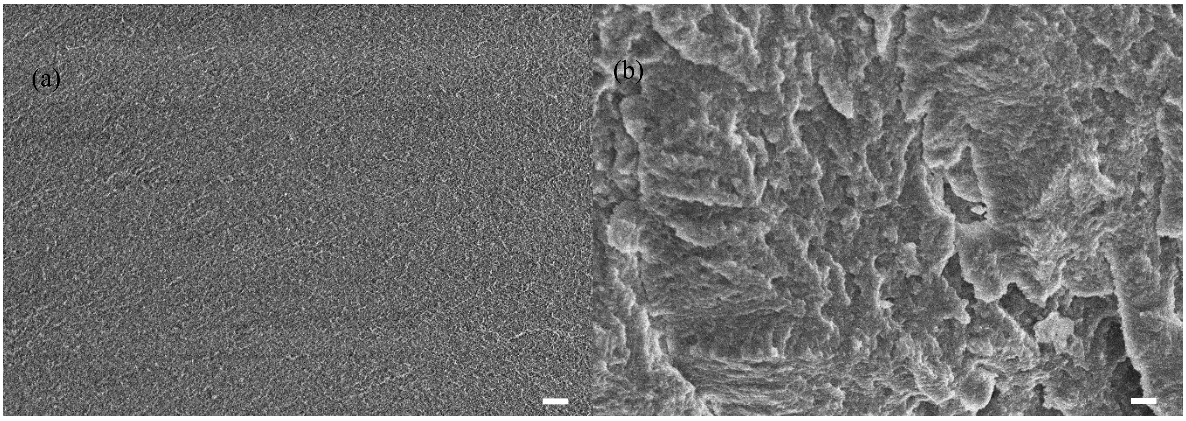

Figure S23: Electron Micrograph (a) PC39 structural battery electrolyte film dried after liquid extraction. (b) PC39 of structural battery electrolyte including liquid electrolyte in cryo-state. Scale bar indicates 1µm

References

(1) Pedersen, J. S. Analysis of Small-Angle Scattering Data from Colloids and Polymer Solutions: Modeling and Least-Squares Fitting. *Adv Colloid Interface Sci* **1997**, *70*, 171–210. https://doi.org/10.1016/S0001-8686(97)00312-6.

(2) Virtanen, P.; Gommers, R.; Oliphant, T. E.; Haberland, M.; Reddy, T.; Cournapeau, D.; Burovski, E.; Peterson, P.; Weckesser, W.; Bright, J.; van der Walt, S. J.; Brett, M.; Wilson, J.; Millman, K. J.; Mayorov, N.; Nelson, A. R. J.; Jones, E.; Kern, R.; Larson, E.; Carey, C. J.; Polat, İ.; Feng, Y.; Moore, E. W.; VanderPlas, J.; Laxalde, D.; Perktold, J.; Cimrman, R.; Henriksen, I.; Quintero, E. A.; Harris, C. R.; Archibald, A. M.; Ribeiro, A. H.; Pedregosa, F.; van Mulbregt, P.; Vijaykumar, A.; Bardelli, A. Pietro; Rothberg, A.; Hilboll, A.; Kloeckner, A.; Scopatz, A.; Lee, A.; Rokem, A.; Woods, C. N.; Fulton, C.; Masson, C.; Häggström, C.; Fitzgerald, C.; Nicholson, D. A.; Hagen, D. R.; Pasechnik, D. V.; Olivetti, E.; Martin, E.; Wieser, E.; Silva, F.; Lenders, F.; Wilhelm, F.; Young, G.; Price, G. A.; Ingold, G.-L.; Allen, G. E.; Lee, G. R.; Audren, H.; Probst, I.; Dietrich, J. P.; Silterra, J.; Webber, J. T.; Slavič, J.; Nothman, J.; Buchner, J.; Kulick, J.; Schönberger, J. L.; de Miranda Cardoso, J. V.; Reimer, J.; Harrington, J.; Rodríguez, J. L. C.; Nunez-Iglesias, J.; Kuczynski, J.; Tritz, K.; Thoma, M.; Newville, M.; Kümmerer, M.; Bolingbroke, M.; Tartre, M.; Pak, M.; Smith, N. J.; Nowaczyk, N.; Shebanov, N.; Pavlyk, O.; Brodtkorb, P. A.; Lee, P.; McGibbon, R. T.; Feldbauer, R.; Lewis, S.; Tygier, S.; Sievert, S.; Vigna, S.; Peterson, S.; More, S.; Pudlik, T.; Oshima, T.; Pingel, T. J.; Robitaille, T. P.; Spura, T.; Jones, T. R.; Cera, T.; Leslie, T.; Zito, T.; Krauss, T.; Upadhyay, U.; Halchenko, Y. O.; Vázquez-Baeza, Y. SciPy 1.0: Fundamental Algorithms for Scientific Computing in Python. *Nat Methods* **2020**, *17* (3), 261–272. https://doi.org/10.1038/s41592-019-0686-2.

(3) Schneider, L. M.; Riazanova, A.; Zenkert, D.; Lindbergh, G. Effect of Electrolyte Composition on Biphasic Structural Electrolytes for Laminated Structural Batteries. *ACS Appl Energy Mater* **2024**, *7* (19), 8838–8850. https://doi.org/10.1021/acsaem.4c01810.
